# Comparative efficacy of pharmacologic interventions in ulcerative colitis: a network meta analysis

**DOI:** 10.1007/s10787-025-01723-z

**Published:** 2025-03-29

**Authors:** Atta Ullah Khan, Maria Ali, Muhammad Aamir Wahab

**Affiliations:** 1 Department of Precision Medicine, University of Campania “Luigi Vanvitelli”, 80138 Naples, Italy; 2 Department of Experimental Medicine, University of Campania “Luigi Vanvitelli”, 80138 Naples, Italy

**Keywords:** Ulcerative colitis, Biologics, Small molecules, Induction, Maintenance

## Abstract

**Introduction:**

Ulcerative colitis is chronic inflammatory condition affecting the colon, necessitating remission inducing therapeutic interventions. With the emergence of newer more advanced options, their relative effectiveness remains unclear. This network meta-analysis (NMA) will compare the effectiveness of presently available biologics and small molecules in achieving and maintaining remission among patients of moderate-to-severe ulcerative colitis as part of induction and maintenance therapy.

**Methods:**

A systematic search was conducted up to 21st February 2025, including only phase 2b/3 or 3 randomized controlled trials. The primary outcome was induction and maintenance of clinical remission (Full Mayo Score (FMS) ≤ 2, with no individual subscore > 1). Secondary outcomes assessed were clinical response, endoscopic improvement (Mayo Endoscopic Score (MES) ≤ 1 either with or without friability) and steroid free remission.

**Results:**

Across 22 studies (7,683 patients), upadacitinib had the highest likelihood of inducing clinical remission (99.08%), clinical response (97.44%) and endoscopic improvement (99.32%), followed by Infliximab and guselkumab following close by for specific outcomes. In maintenance of clinical remission and endoscopic improvement upadacitinib again ranked highest (95.60%) and (99.46%). Tofacitinib (92.43%) has the highest probability with upadacitinib (87.73%) following behind in achieving steroid free remission.

**Conclusion:**

Upadacitinib displayed high efficacy across multiple outcomes in both induction and maintenance therapy with Infliximab, guselkumab, and filgotinib following closely behind. For achieving steroid free remission tofacitinib has the highest probability of doing so. Overall small molecules and selective IL-23 inhibitors seems promising alternative to older biologics though additional head-to-head trial are warranted along with more real-world data.

**Supplementary Information:**

The online version contains supplementary material available at 10.1007/s10787-025-01723-z.

## Introduction

Ulcerative colitis (UC) is among the two well-known forms of inflammatory bowel disease (IBD), characterized by a continuously progressing inflammation that starts from rectum moving proximally affecting the mucosa. Ulcerative colitis (UC) follows a relapsing-remitting course and with the global prevalence on the rise UC poses a significant impact on patients’ quality of life (Caron et al. 2024; Feuerstein and Cheifetz 2014; Lewis et al. 2023). The exact cause of UC development is still far from being fully known but factors believed to be associated with its development or somehow play a part in its development includes genetic, environmental and at the forefront is a dysregulated immune response (Danese and Fiocchi 2011; Kobayashi et al. 2020; Voelker 2024). The fundamental goal of any UC treatment is the reduction in symptoms and maintenance of that state, preventions of complications usually associated with UC and finally improve patient quality of life (Raine et al. 2022; Rubin et al. 2019). The usual medications for UC includes 5-aminosalicylates (5-ASA), corticosteroids, and immunomodulators as first line therapy. These are vital in restricting disease progression, but a more specific approach is certainly needed considering the wide-ranging side effects associated with these medications.

With that in mind, a new class of drugs called biologics which at molecular level comprises of monoclonal antibodies that target multiple pathway components involved in the inflammatory response has been developed. These include tumor necrosis factor (TNF-α) inhibitors, such as infliximab and adalimumab which were among the first biologics to gain approval and show efficacy in both inducing and maintaining remission (D’Amico et al. 2020; Danese et al. 2015; Fausel et al. 2015). Moving forward, the development of newer biologics, such as anti-integrins (e.g., vedolizumab) as well as interleukin antagonists (e.g., ustekinumab) have been achieved, thereby expanding the already extensive set of therapy choices for UC (Hanžel and D’Haens 2020; Kashani and Schwartz 2019; Lamb et al. 2018; Thomas and Baumgart 2012).

The latest class of medication available for UC therapy and that also have gained FDA approval includes Janus kinase (JAK) inhibitors and sphingosine 1-phosphate (S1P) modulators. These are known as small molecules drugs that also offer the convenience of oral administration. Janus kinase (JAK) inhibitors target numerous molecules belonging to the JAK-STAT signaling pathway while sphingosine 1-phosphate (S1P), a lipid-based mediator involved in immune trafficking and vascular health. They provide more robust immunosuppressive effect and have shown efficacy in both induction of and maintaining remission in UC patients (Ben Ghezala et al. 2021; Jiang et al. 2022; Liang et al. 2024; Nakamura et al. 2024; Neri et al. 2024; Shivaji et al. 2020; Vieujean et al. 2025).

With increasing diversity in therapeutic options becoming available, the need for comparative efficacy and safety evaluations is becoming more important today to specifically evaluate and synthesize the available evidence on clinically meaningful endpoints that reflect key therapeutic goals, including clinical remission, clinical response, and endoscopic improvement during induction therapy, as well as clinical remission, endoscopic improvement, and steroid-free remission during maintenance therapy to guide clinical judgment. While standard meta-analyses do hold value the heterogeneity in patient populations, disease severity, and treatment protocols across studies highlights the importance of a network-based approach that can integrate diverse sources of evidence. Network meta-analysis (NMA) removes these limitations by allowing the indirect comparison of many therapies using a common comparator and establishing a treatment hierarchy based on their relative efficacy and safety.

The aim of this NMAis to compare the efficacy of biologics and small molecules utilizing key outcomes as part of both induction and maintenance therapy.

## Methods

### Search strategy and selection criteria

This Network Meta Analysis adheres to the Preferred Reporting Items for Systematic Reviews and Meta-Analyses for Network Meta-Analyses (PRISMA-NMA) guidelines (Hutton et al. 2015). The protocol was registered with the International Prospective Register of Systematic Reviews (PROSPERO) under registration number CRD420250655739.

A wide-ranging retrieval strategy was developed to catch all relevant studies. From inception to 21 January 2025, two independent reviewers (MAW and MA) performed a very thorough search of search of PubMed, Embase, Web of Science and Cochrane Central Register of Controlled Trials (CENTRAL). Concomitantly, unpublished or advancing studies were also searched by reviewing multiple leading gastroenterology symposiums (e.g., ECCO) and clinical trial registers (e.g., ClinicalTrials.gov).

Search terms included Medical Subject Headings (MeSH) and free-text keywords related to ulcerative colitis (UC), biologics, small molecules, and efficacy outcomes. Specific search strings combined terms such as “ulcerative colitis,” “biologics,” “small molecules,” “clinical remission,” “endoscopic improvement,” and drug-specific names (e.g., infliximab, vedolizumab, tofacitinib, upadacitinib). No language or geographic restrictions were applied. Detailed search strategy is available in Supplementary Table 1.

Randomized control trials (RCTs) that are Phase 2b/3 or 3 evaluating biologics or small molecule drugs against placebo or other active comparator involving adults (≥18 years) with a confirmed diagnosis of moderate-to-severe UC defined by a Full Mayo Score (MCS) of 6–12 (Dignass et al. 2012; Sicilia et al. 2020). Studies reporting efficacy outcomes, including clinical remission, clinical response, endoscopic improvement, and steroid-free remission were included.

RCTs that are not phase 2b/3 or 3. Studies focusing on pediatric populations. Trials that do not report key efficacy outcomes according to defined criteria. Studies about non-human subjects or in vitro testing, non-randomized studies, observational studies, case reports, reviews, summaries, meta-analyses, letters to the editor along with irretrievable full text studies or with incomplete data were excluded.

#### Outcomes

Induction therapy was considered for 6–14 weeks, outcomes assessed were clinical remission (defined as FMS ≤2, with no individual subscore >1), clinical response (defined as Reduction in FMS by ≥3 points and ≥30% from baseline, including a decrease in rectal bleeding subscore) and endoscopic improvement (defined as MES ≤1 either with or without friability). The maintenance Phase was considered for ≥40 weeks and the assessed outcomes were clinical remission, endoscopic improvement and steroid-free remission.

To maintain uniformity across studies, outcomes for mucosal healing and endoscopic improvement were harmonized as much as possible based on their definitions even though studies reported these outcomes under different terminologies, they were included in the same analytical category if they shared identical definitions which is Mayo Endoscopic Subscore (MES) of ≤1.

#### Study selection

Two independent reviewers (MAW and MA) extracted data from eligible studies using a standardized data extraction form. Extracted information included, study related information (author, year), patient-related information (age, sex, disease duration), intervention-related details (drug, dosage, administration route) and outcome measures (time points, and results for each efficacy endpoint).

Discrepancies in data extraction were resolved through consensus or consultation with a third reviewer.

#### Risk of Bias and quality assessment

The Cochrane Risk of Bias 2.0 tool (RoB 2) (Sterne et al. 2019), was used to assess the methodological quality of included RCTs. This tool evaluates studies across five domains, Bias arising because of the randomization process, Bias arising as a result of deviation from intended interventions, Bias due to lack of outcome data, Bias due to measurement of the outcome and Bias because of selection of the reported result. Individual studies were classified as having a low, some concerns, or high risk of Bias based on individual domain evaluation. For results of RoB assessment see Supplementary Figure S1. Discrepancies were resolved through discussion or by consultation involving a third reviewer.

#### Statistical analysis

A frequentist approach was utilized to conduct our NMA using R software version 4.4.2 and netmeta package (Balduzzi et al. 2023). Effect measures comprising of odds ratios with 95% Confidence Intervals (95% CI) were calculated utilizing dichotomous data. *I*^2^ statistic was utilized for calculating in between studies heterogeneity (Higgins et al. 2003; Higgins and Thompson 2002), node splitting model used for inconsistency determination. Lastly P-Scores was used to rank treatments efficacy with higher scores denoting better efficacy.

## Results

**Fig. 1:**
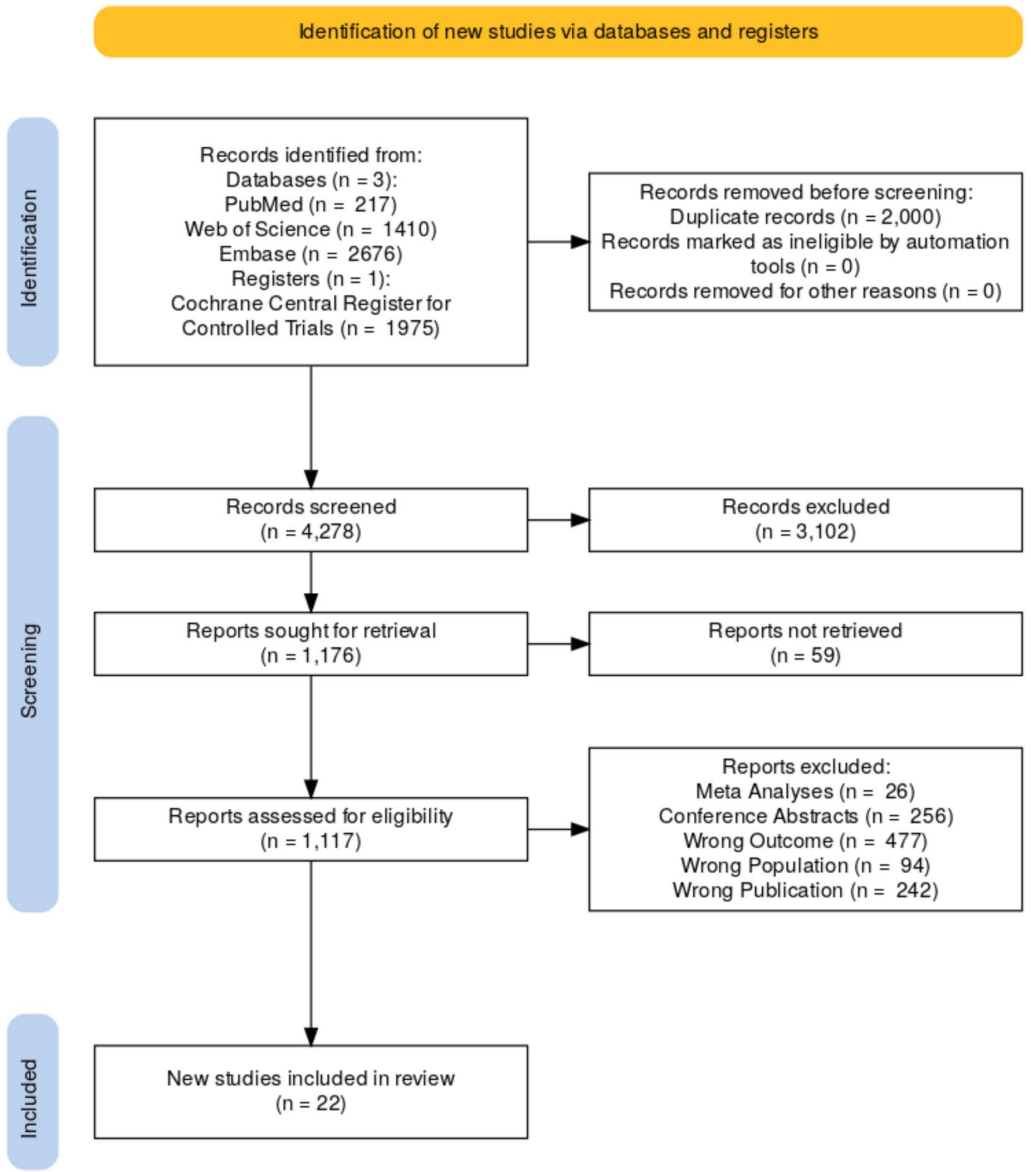
PRISMA flow chart of search strategy

A total of 22 randomized controlled trials (RCTs), comprising of 7,683 patients with moderate-to-severe UC Fig. 1, were included in this NMA (Danese, Colombel, et al., 2022; Danese, Vermeire, et al., 2022; D’Haens et al. 2023; Feagan et al. 2013, 2021; Hibi et al. 2017; Jiang et al. 2015; Motoya et al. 2019; Panaccione et al. 2014; Peyrin-Biroulet et al. 2022; Reinisch et al. 2011; Rubin et al. 2024; Rutgeerts et al. 2005; Sandborn et al. 2012, 2017, 2020, 2021; Sandborn et al. 2014; Sandborn et al. 2014a, b; Sands et al. 2019a, b; Sands et al. 2019a, b; Suzuki et al. 2014). The studies evaluated the efficacy of biologics and small molecules, including Upadacitinib, Infliximab, Ozanimod, Guselkumab, Ustekinumab, Tofacitinib, Golimumab, Etrolizumab, Vedolizumab, Filgotinib, Mirikizumab, and Adalimumab, compared to placebo. The interventions were assessed for their effectiveness in inducing remission, response, and endoscopic improvement and maintaining remission, endoscopic improvement and steroid free remission. Baseline characteristics of included studies are presented in Supplementary Table 2. Heterogeneity was low in all the analyzed outcomes for both induction and maintenance therapies, with *I*^2^ < 30%, and there were no elements for inconsistency (*p* < 0.05) invalidating the model. The results are represented in the supplementary appendix.

### Induction therapy

#### Clinical remission

**Fig. 2:**
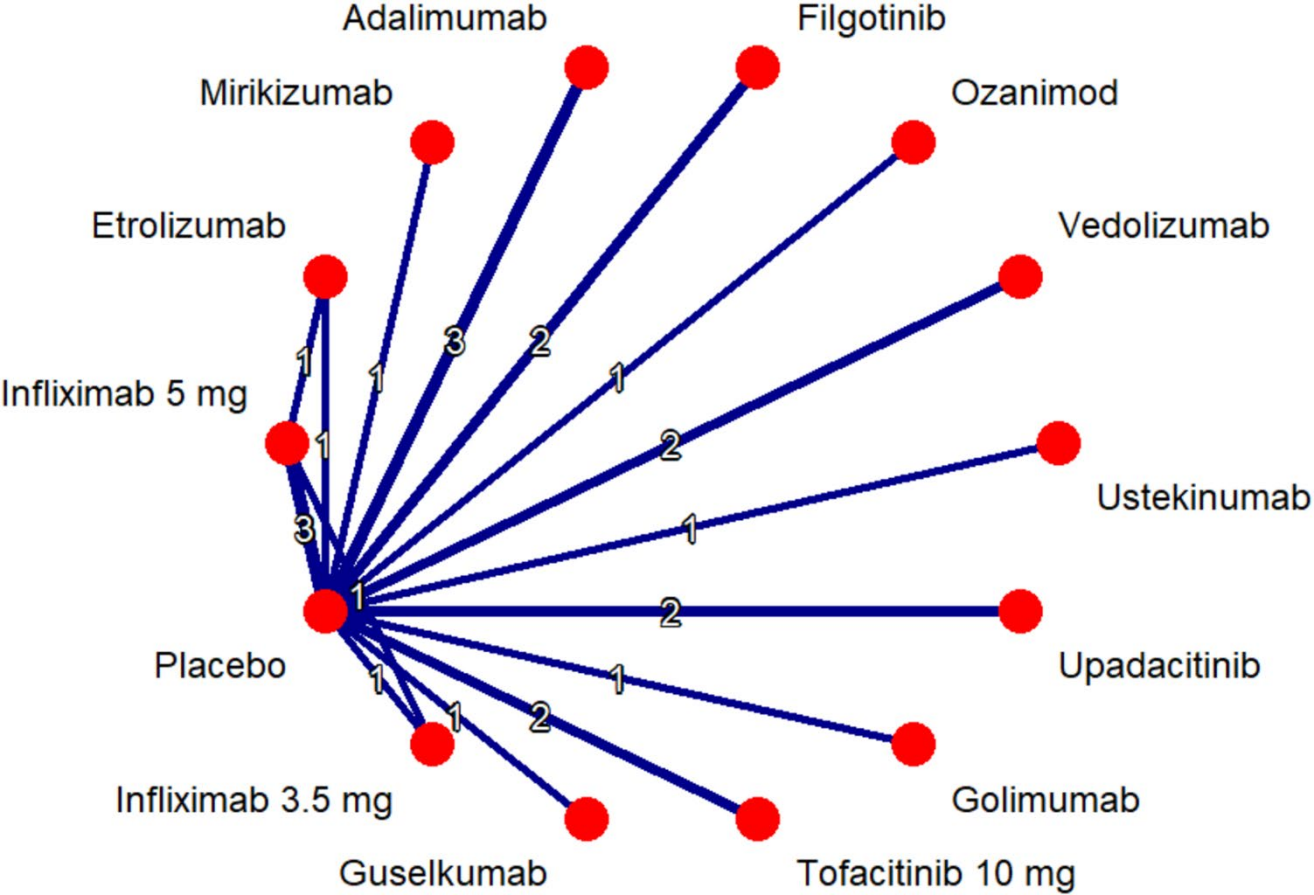
Network plot for clinical remission—induction therapy

All treatments displayed statistical significance in inducing clinical remission as compared to placebo with upadacitinib (OR 9.43, 95% CI 5.36–16.61), infliximab 5 mg (OR 4.96, 95% CI 3.29–7.46) and infliximab 3.5 mg (OR 4.17, 95% CI 1.84–9.41) ranking 1st, 2nd and 3rd. In comparison to other treatments upadacitinb possessed statistically significant superiority over all except infliximab 5 mg (OR 1.90, 95% CI 0.95–3.82) and infliximab 3.5 mg (OR 2.26, 95% CI 0.84–6.11). In addition, infliximab 5 mg showed statistically significant superiority over etrolizumab, vedolizumab, filgotinib, mirikizumab and adalimumab as is evident from Suplementary Table 3. Per the P-score ranking, upadacitinib ranked highest in inducing clinical remission (99.08%), followed by Infliximab 5 mg (84.6%). Infliximab 3.5 mg (70.93%) ranked third. P-score table is available as part of Supplementary Figure S2. Also, for network plot see Fig. 2.

#### Clinical response

All treatments displayed statistical significance in inducing clinical response as compared to placebo except for azathioprine (OR 1.78, 95% CI 0.79–4.01). Upadacitinib (OR 7.85, 95% CI 5.41–11.38), infliximab/azathioprine (OR 5.94, 95% CI 2.53–13.98) and guselkumab (OR 4.14, 95% CI 2.64–6.49) ranking 1st, 2nd and 3rd when compared to placebo. In comparison to other treatments upadacitinb statistically significant superiority over all except infliximab/azathioprine (OR 1.32, 95% CI 0.52–3.35) and infliximab 3.5 mg (OR 2.01, 95% CI 0.76–5.32) as is eveident from Suplementary Table 4. Per the P-score ranking, upadacitinib ranked highest in inducing clinical response (97.44%), followed by infliximab/azathioprine (87.79%) and guselkumab (76.08%) ranked third. P-score table is available as part of Supplementary Figure S3.

#### Endoscopic improvement

All treatments displayed statistical significance in inducing endoscopic improvement as compared to placebo except for azathioprine (OR 1.55, 95% CI 0.76–3.17). Upadacitinib (OR 8.25, 95% CI 5.33–12.77), infliximab/azathioprine (OR 4.49, 95% CI 2.21–9.14) and infliximab 5 mg (OR 3.19, 95% CI 2.35–4.33) ranked 1st, 2nd and 3rd. In comparison to other treatments, upadacitinib showed statistical significance over all except infliximab/azathioprine as is evident from Suplementary Table 5. Per the P-score ranking, upadacitinib ranked highest in inducing endoscopic improvement (99.32%), followed by Infliximab/azathioprine (87.17%) while infliximab 5 mg (75.89%) ranked third. P-score table is available as part of Supplementary Figure S4.

### Maintenance therapy

#### Clinical remission

**Fig. 3:**
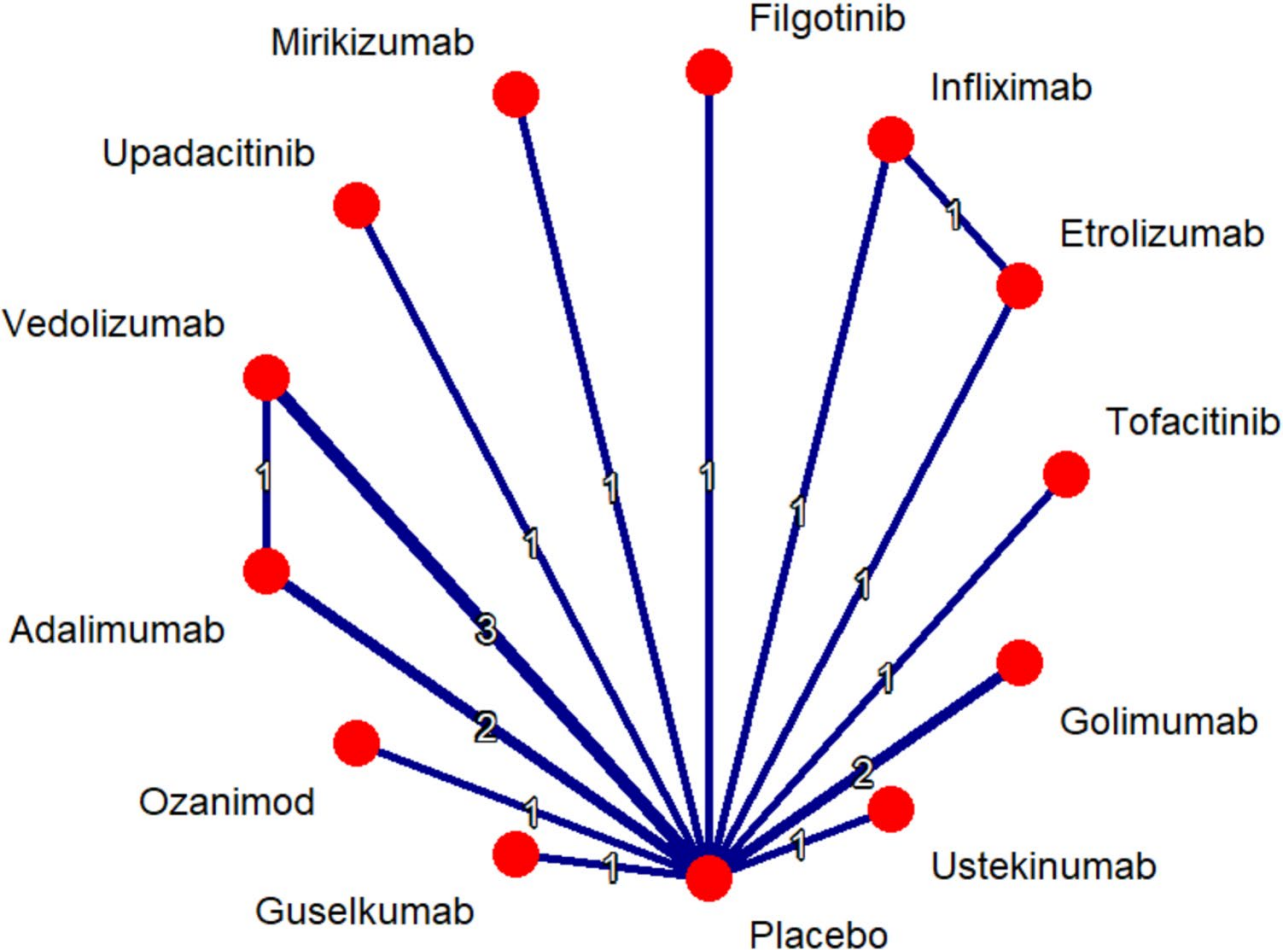
Network plot clinical remission- maintenance therapy

All treatments except for etrolizumab showed statistical significance in maintaining clinical remission in comparison to placebo. Upadacitinib (OR 7.87, 95% CI 3.90–15.88) displayed statistically significant superiority to all other treatments except filgotinib, guselkumab, tofacitinib and vedolizumab as is evident from Suplementary Table 6. According to P- Score, upadacitinib (95.60%) ranked highest in maintaining clinical remission with filgotinib (81.28%) and guselkumab (73.75%) following behind, P-score table is available as part of Supplementary Figure S5. As for network plot see Fig. 3.

#### Endoscopic improvement

All treatments were significantly superior in comparision to placebo for maintaining endoscopic improvement. Except for guselkumab, upadacitinib was significantly superior to all drugs in maintaining endoscopic improvement. Upadacitinib (OR 9.30, 95% CI 5.32–16.23), guselkumab (OR 4.56, 95% CI 2.87–7.23) and tofacitinib (OR 3.95, 95% CI 2.39–6.53) ranked 1st, 2nd and 3rd as seen through Supplementary Table 7. P-scores ranking also confirms upadacitinib (99.46%) ranked highest in maintaining endoscopic improvement with guselkumab (80.46%) and tofacitinib (70.67%) following behind. P-score table is available as part of Supplementary Figure S6.

#### Steroid free remission

With the exception of golimumab all treatments were significantly superior in comparison to placebo in achieving steroid free remission. Tofacitinib (OR 10.22, 95% CI 2.88–36.32) has the best odds in achieving steroid free remission with upadacitinib (OR 7.18, 95% CI 3.09–16.71) and adalimumab (OR 4.51, 95% CI 2.31–8.79) coming 2nd and 3rd. Looking at P-scores we can see that tofacitinib (92.43%) has the highest probability with upadacitinib (87.73%) and adalimumab (71.59%) following behind as is evident from Suplementary Table 8. P-score table is available as part of Supplementary Figure S7.

## Discussion

A comprehensive comparative assessment of treatment options comprising of biologics and small molecules available for moderate-to-severe UC treatment through this NMA was carried out revealing upadacitinib demonstrating superior efficacy in inducing clinical remission, clinical response, and endoscopic improvement, as well as maintaining remission and endoscopic improvement, this positions Janus kinase (JAK) inhibitors such as upadacitinib and tofacitinib in particular, as an effective option in both induction and maintenance therapies. Infliximab an anti-TNF agent also gave performed well in inducing remission and endoscopic improvement, following just behind upadacitinib. Likewise, guselkumab, an IL-23 inhibitor, also performed well in achieving clinical response and maintaining endoscopic improvement. Tofacitinib exhibited the highest probability of achieving steroid-free remission, reinforcing the relevance of JAK inhibitors in reducing corticosteroid dependence in UC management. Filgotinib, which is another member of the JAK inhibitor family of medications, was also quite impressive during maintenance therapy, particularly in sustaining clinical remission, which suggests that selective JAK1 inhibition might also be an effective and well-tolerated option for long-term UC management. The S1P receptor modulator ozanimod did perform well but its comparative effectiveness did not surpass those of JAK inhibitors and IL-23 antagonists, which indicates that newer biologics might be preferable in many cases.

The results of this NMA emphasizes the importance of personalized treatment strategies integrating patient specific factors including previous medication history of patients. Keeping that in mind results from this NMA also reinforces the fact that IL-23 inhibitors could be a viable alternative therapeutic option with the strong performance of guselkumab in clinical response and maintenance of endoscopic improvement further supports the integration of IL-23 antagonists into treatment algorithms for UC.

## Conclusion

While the results from our NMA does provide a robust comparative efficacy ranking of currently available treatment options for UC management, several undeniable limitations still exists that need acknowledgment. First, real-world performance may be different from trial setting. Second, data on safety outcomes were excluded from this NMA mainly because of too much variability precluding any meaningful harmonization. As a result, a definitive conclusion regarding which treatment is both effective and safe is not possible here. Future studies with standardized safety data are needed to carry out a comprehensive benefit-risk profile of treatments for UC

According to this NMA upadacitinib came out to be the most effective therapy across multiple outcomes following close behind are infliximab, guselkumab and tofacitinib outranking upadacitinib in specific outcomes. JAK inhibitors and IL-23 inhibitors emerge as promising alternatives due to their strong efficacy. Future research should focus on identifying biomarkers for response prediction and optimizing treatment sequencing to improve patient outcomes in UC management.

## Supplementary Information

Below is the link to the electronic supplementary material.

Supplementary file1 (DOCX 413 KB)

## Data Availability

The datasets generated during and/or analyzed during the current study are available from the corresponding author on reasonable request.
