# Supplementary material for: Comparative efficacy of pharmacologic interventions in ulcerative colitis: a network meta analysis
Source: Inflammopharmacology. 2025 Mar 29;33(5):2679–87. doi: 10.1007/s10787-025-01723-z (PMC12176966; doi:10.1007/s10787-025-01723-z)
Supplement: Supplementary file 1 — Supplementary file1 (DOCX 413 KB) [file 10787_2025_1723_MOESM1_ESM.docx]

**Supplementary Data**

Supplementary Table 1. Detailed Search strategy

| Database | Keywords | Results |
| --- | --- | --- |
| PubMed | (Inflammatory Bowel Disease OR  colitis, ulcerative [MeSH] OR  ulcerative colitis OR (ulcerative AND colitis) | 149,124 |
|  | (Biologics OR biological products [MeSH] OR small molecules*[MeSH] OR “induction"[All  Fields] OR “maintenance"[All Fields] OR anti-TNF OR " tumor necrosis factor inhibitors "[All  Fields] OR infliximab OR adalimumab OR golimumab OR anti-integrin OR  vedolizumab OR etrolizumab OR interleukin 23 antagonist [MeSH] OR ustekinumab OR  mirikizumab OR guselkumab) OR (risankizumab) OR (JAK inhibitor [MeSH] OR tofacitinib OR filgotinib OR upadacitinib OR sphingosine-1-phosphate receptor modulator OR ozanimod OR etrasimod) | 8,861,055 |
|  | remissions OR endoscopic OR remission OR mucosal) AND healing OR normalization OR  improvement AND steroid free remission AND Randomized Controlled Trials | 1,283 |
|  | #1 AND #2 AND #3 | 217 |
| Web of Science | Inflammatory Bowel Disease OR colitis, ulcerative OR ulcerative colitis OR ulcerative AND colitis AND biologics OR small molecules OR induction OR maintenance OR anti-TNF OR tumor necrosis factor inhibitors OR infliximab OR adalimumab OR golimumab OR anti-integrin OR vedolizumab OR etrolizumab OR interleukin 23 antagonist OR ustekinumab OR mirikizumab OR guselkumab OR risankizumab OR JAK inhibitor OR tofacitinib OR filgotinib OR upadacitinib OR sphingosine-1-phosphate receptor modulator OR ozanimod OR etrasimod AND response OR remission OR mucosal healing OR endoscopic improvement OR steroid free remission AND Randomized Controlled Trials | 1,410 |
| Embase | Inflammatory Bowel Disease OR colitis, ulcerative OR ulcerative colitis OR ulcerative AND colitis AND biologics OR small molecules OR induction OR maintenance OR anti-TNF OR tumor necrosis factor inhibitors OR infliximab OR adalimumab OR golimumab OR anti-integrin OR vedolizumab OR etrolizumab OR interleukin 23 antagonist OR ustekinumab OR mirikizumab OR guselkumab OR risankizumab OR JAK inhibitor OR tofacitinib OR filgotinib OR upadacitinib OR sphingosine-1-phosphate receptor modulator OR ozanimod OR etrasimod AND response OR remission OR mucosal healing OR endoscopic improvement OR steroid free remission AND Randomized Controlled Trials | 2676 |
| Cochrane Central Register for Controlled Trials | Inflammatory Bowel Disease OR colitis, ulcerative OR ulcerative colitis OR ulcerative AND colitis in Title Abstract Keyword AND biologics OR small molecules OR induction OR maintenance OR anti TNF OR tumor necrosis factor inhibitors OR infliximab OR adalimumab OR golimumab OR anti integrin OR vedolizumab OR etrolizumab OR interleukin 23 antagonist OR ustekinumab OR mirikizumab OR guselkumab OR risankizumab OR JAK inhibitor OR tofacitinib OR filgotinib OR upadacitinib OR sphingosine 1 phosphate receptor modulator OR ozanimod OR etrasimod in Title Abstract Keyword AND response OR remission OR mucosal healing OR endoscopic improvement OR steroid free remission AND Randomized Controlled Trials | 1975 |

Supplementary Table 2. Baseline Characteristics of Included Studies

| Study | Intervention | Dosing | Male  [n (%)] | Age (years) [Mean ± SD] or [Mean; range] | Disease duration (years) [Mean±SD] or [Mean; range] | Mayo score [Mean±SD] or [Mean; range] | Concomitant medications, n (%) | | |
| --- | --- | --- | --- | --- | --- | --- | --- | --- | --- |
|  |  |  |  |  |  |  | Corticosteroids | 5-aminosalicylates | Immunosuppressants  /Immunomodulators |
| ULTRA 1  [NCT00385736] | ADA | 160/80/40 mg SC | 63.8% | 36.5 (18-75) | 6.06 (0.2-34.4) | 8.8±1.61 | 48 (36.9) [only]  23 (17.7) [+immunomodulators] | 105 (80.8) | 28 (36.9) [only] |
|  | PLC | - | 63.1% | 37 (18-72) | 5.35 (0.3-34.1) | 8.7±1.56 | 55 (41.5) [only]  34 (26.1) [+immunomodulators] | 98 (76.2) | 18 (13.8) [only] |
| ULTRA 2  [NCT00408629] | ADA | 160/80 mg | 142 (57.3) | 39.6±12.47 | 8.1±7.09 | 8.9±1.50 | 150 (60.5) | 146 (58.9) | 93 (37.5) |
|  | PLC | - | 152 (61.8) | 41.3±13.22 | 8.5±7.37 | 8.9±1.75 | 140 (56.9) | 155 (63.0) | 80 (32.5) |
| Suzuki 2014 | ADA | 160/80/40 mg SC | 61 (67.8) | 42.5±14.6 | 7.8±7.1 | 8.6±1.4 | 57 (53.3) | 83 (92.2) | 41 (45.6) |
|  | PLC | - | 70 (72.9) | 41.3±13.6 | 7.8±6.6 | 8.5±1.6 | 58 (60.4) | 89 (92.7) | 52 (54.2) |
| PURSUIT-SC  [NCT00487539] | GLM | 200/100 mg SC | 140 (54.3) | 39.7±13.79 | 6.4±6.42 | 8.6±1.53 | 142 (42.9) [excl. budesonide] | 270 (81.6) | 105 (31.7) |
|  | PLC | - | 130 (50.4) | 39.7±13.35 | 6.4±7.23 | 8.3±1.50 | 134 (40.5) [excl. budesonide] | 276 (83.4) | 106 (32.0) |
| PURSUIT-M  [NCT00488631] | GLM | 100 mg SC | 89 (57.8) | 39.1±13.11 | 7.2±7.04 | 8.5±1.34 | 79 (51.3) [excl. budesonide] | 119 (77.3) | 48 (31.2) |
|  | PLC | - | 75 (48.1) | 40.2±14.05 | 6.9±6.96 | 8.3±1.37 | 83 (53.2) [excl. budesonide] | 125 (80.1) | 52 (33.3) |
| ACT 1  [NCT00036439] | INF | 5 mg/kg IV | 78 (64.5) | 42.4±14.3 | 5.9±5.4 | 8.5±1.7 | 70 (57.9) | 82 (67.8) | 66 (54.5) |
|  | PLC | - | 72 (59.5) | 41.4±13.7 | 6.2±5.9 | 8.4±1.8 | 79 (65.3) | 85 (70.2) | 53 (43.8) |
| ACT 2  [NCT00096655] | INF | 5 mg/kg IV | 76 (62.8) | 40.5±13.1 | 6.7±5.3 | 8.3±1.5 | 60 (49.6) | 92 (76.0) | 52 (43.0) |
|  | PLC | - | 71 (57.7) | 39.3±13.5 | 6.5±6.7 | 8.5±1.5 | 60 (48.8) | 89 (72.4) | 54 (43.9) |
| Jiang 2015 | INF | 3.5 mg/kg IV | 24 (58.5) | 34.1±13.8 | 4.3±2.5 | 6.0 (5.0-6.0)* | 22 (53.7) | 36 (87.8) | 12 (29.3) |
|  | INF | 5 mg/kg IV | 26 (63.4) | 34.3±14.3 | 4.4±2.8 | 6.0 (5.0-6.0)* | 22 (53.7) | 34 (82.9) | 12 (29.3) |
|  | PLC | - | 25 (60.9) | 34.5±14.9 | 4.4±2.6 | 6.0 (5.0-6.0)* | 21 (51.2) | 35 (85.4) | 13 (31.7) |
| LUCENT-1  [NCT03518086] | MIC | 300 mg IV | 530 (61.1) | 42.9±13.9 | 7.2±6.7 | 4-9 | 351 (40.4) | 646 (74.4) | 211 (24.3) |
|  | PLC | - | 165 (56.1) | 41.3±13.8 | 6.9±7.0 | 4-9 | 113 (38.4) | 217 (73.8) | 69 (24.3) |
| LUCENT-2  [NCT03524092] | MIC | 200 mg SC | 214 (58.6) | 43.4±14.2 | 6.9±7.1 | - | 135 (37.0) | 278 (76.2) | 78 (21.4) |
|  | PLC | - | 104 (58.1) | 41.2±12.8 | 6.7±5.6 | - | 68 (38.0) | 134 (74.9) | 39 (21.8) |
| UNIFI  [NCT02407236] | UST | 6 mg/kg IV | 195 (60.6) | 41.7±13.7 | 8.2±7.8 | 8.9±1.5 | 168 (52.2) | 238 (73.9) | 89 (27.6) |
|  | PLC | - | 197 (61.8) | 41.2±13.5 | 8.0±7.2 | 8.9±1.6 | 157 (49.2) | 207 (64.9) | 89 (27.9) |
| GEMINI 1  [NCT00783718] | VED | 300 mg IV | 132 (58.7) | 40.1±13.1 | 6.1±5.1 | 8.5±1.8 | 79 (35.1) [only]  47 (20.9) [+immunosuppressants] | - | 28 (12.4) [only] |
|  | PLC | - | 92 (61.7) | 41.2±12.5 | 7.1±7.2 | 8.6±1.7 | 58 (38.9) [only]  26 (17.4) [+immunosuppressants] | - | 18 (12.1) [only] |
| Motoya 2019  [NCT02039505] | VED | 300 mg IV | 99 (60.4) | 42.3 (14.4) | 7.2 (6.2) | 8.3±1.5 | 31 (18.9) [only oral] | 145 (88.4) | 59 (36.0) [only] |
|  | PLC | - | 55 (67.1) | 44.0 (16.0) | 8.6 (8.0) | 8.1±1.5 | 11 (13.4) [only oral] | 75 (91.5) | 29 (35.4) [only] |
| VARSITY  [NCT02497469] | ADA | 160/80 mg SC | 216 (56.0) | 40.5±13.4 | 6.4±6.0 | 8.7±1.5 | 140 (36.3) [only] | - | 100 (25.9) [only] |
|  | VED | 300 mg IV | 234 (60.8) | 40.8±13.7 | 7.3±7.2 | 8.7±1.6 | 139 (36.1) [only] | - | 101 (26.2) [only] |
| U–ACHIEVE | PLC | - | 97 (63%) | 44·5 (23·0) | 6·0 (10·0) | - | 61 (40%) | 103 (67%) | 3 (2%) |
|  | UPA | 45 mg PO | 198 (62%) | 43·0 (23·0) | 6·6 (9·6) | - | 124 (39%) | 220 (69%) | 2 (1%) |
| U–ACCOMPLISH | PLC | - | 107 (61%) | 42·0 (24·0) | 4·9 (7·4) | - | 72 (41%) | 120 (69%) | 3 (2%) |
|  | UPA | 45 mg PO | 214 (63%) | 40·0 (24·0) | 5·6 (7·5) | - | 120 (35) | 233 (68%) | 1 (<1%) |
| U–ACHIEVE | PLC | - | 85 (57%) | 40·0 (21·0) | 6·2 (8·6) | - | 60 (40%) | 99 (66%) | 0 |
|  | UPA | 30 mg PO | 86 (56%) | 41·0 (7·0) | 6·0 (9·7) | - | 57 (37%) | 106 (69%) | 1 (<1%) |
| SUCCESS | AZA | 2.5 mg PO | 33 (42%) | 40.7 (13.2) | 6.6 (7.8) | 8.5 (1.4) | 27 (34.2) | - | 8 (10.1) |
|  | INF | 5 mg/kg IV | 42 (54%) | 38.5 (12.7) | 6.3 (6.5) | 8.1 (1.4) | 31 (39.7) | - | 8 (10.3) |
|  | INF/AZA | - | 48 (60%) | 38.0 (12.2) | 5.2 (5.1) | 8.6 (1.3) | 38 (47.5) | - | 8 (10.0) |
| GARDENIA | ETR | 105 mg SC | 118 (59%) | - | - | 8.60 (1∙53) | 91 (46%) | 158 (79%) | 68 (34%) |
|  | INF | 5 mg/kg IV | 132 (67%) | - | - | 8.59 (1∙52) | 94 (47%) | 168 (85%) | 68 (34%) |
| HICKORY | PLC | - | 54 (57%) | - | - | 9.02 (1∙51) | 45 (47%) | 52 (55%) | 27 (28%) |
|  | ETR | 105 mg SC | 224 (58%) | - | - | 8.95 (1∙61) | 182 (47%) | 232 (60%) | 112 (29%) |
| VISIBLE 1 | PLC | - | 34 (60.7) | 39.4 (11.7) | 7.4 (7.1) | - | 24 (42.9) | - | - |
|  | VED | 300 mg IV | 31 (57.4) | 41.6 (14.1) | 8.2 (5.9) | - | 21 (38.9) | - | - |
| OCTAVE I | PLC | - | 77 (63.1) | 41.8±15.3 | - | 9.1±1.4 | 58 (47.5) | - | - |
|  | TOF | 10 mg PO | 277 (58.2) | 41.3±14.1 | - | 9.0±1.4 | 214 (45.0) | - | - |
| OCTAVE I | PLC | - | 55 (49.1) | 40.4±13.2 | - | 8.9±1.5 | 55 (49.1) | - | - |
|  | TOF | 10 mg PO | 259 (60.4) | 41.1±13.5 | - | 9.0±1.5 | 198 (46.2) | - | - |
| SELECTION A | PLC | - | 87 (63.5%) | 41 (12·9) | 6·4 (7·4) | 8·7 (1·3) | 34 (24·8%) | - | 33 (24·1%) |
|  | FIL | 200 mg PO | 123 (50.2%) | 42 (13·1) | 7·2 (6·9) | 8·6 (1·3) | 54 (22·0%) | - | 53 (21·6%) |
| SELECTION B | PLC | - | 86 (60·6%) | 44 (14·9) | 10·2 (8·2) | 9·3 (1·4) | 51 (35·9%) | - | 21 (14·8%) |
|  | FIL | 200 mg PO | 148 (56·5%) | 43 (14·2) | 9·8 (7·6) | 9·2 (1·4) | 94 (35·9%) | - | 34 (13·0%) |
| QUASAR | PLC | - | 161 (58%) | 39·8 (13·4) | 7·1 (6·5) | 9·2 (1·3) | 120 (49%) | 204 (83%) | 54 (22%) |
|  | GUS | 200 mg IV | 238 (57%) | 41·0 (13·9) | 7·8 (7·7) | 9·1 (1·4) | 182 (50%) | 304 (83%) | 92 (25%) |
| TRUE NORTH | PLC | - | 143 (66.2) | 41.9±13.6 | 6.8±7.0 | 8.9±1.4 | 70 (32.4) | 182 (84.3) | 13 (6.0) |
|  | OZA | 1 mg PO | 245 (57.1) | 41.4±13.5 | 6.9±6.6 | 8.9±1.5 | 119 (27.7) | 374 (87.2) | 19 (4.4) |
| PERSUIT J | PLC | - | 19 (61%) | 42.90 (14.41) | - | - | 9 (29%) | 27 (87%) | - |
|  | GLM | 100 mg SC | 19 (59%) | 39.30 (12.00) | - | - | 9 (28%) | 29 (91%) | - |

ADA – adalimumab; GLM – golimumab; INF – infliximab; MIC – mirikizumab; UST – ustekinumab; VDZ – vedolizumab; GUS – guselkumab; FIL – filgotinib; TOF – tofacitinib; ETR – etrolizumab; AZA – azathioprine; UPA – upadacitinib; OZA - ozanimod

Supplementary Figure S1. Risk of Bias Assessment based on Cochrane risk of bias assessment tool 2.0.

A B


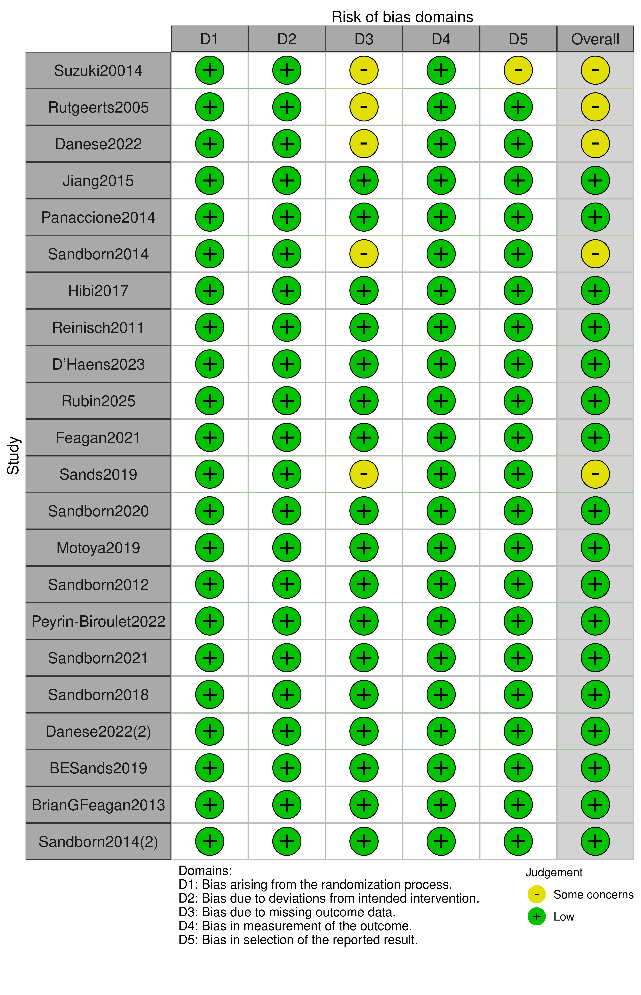


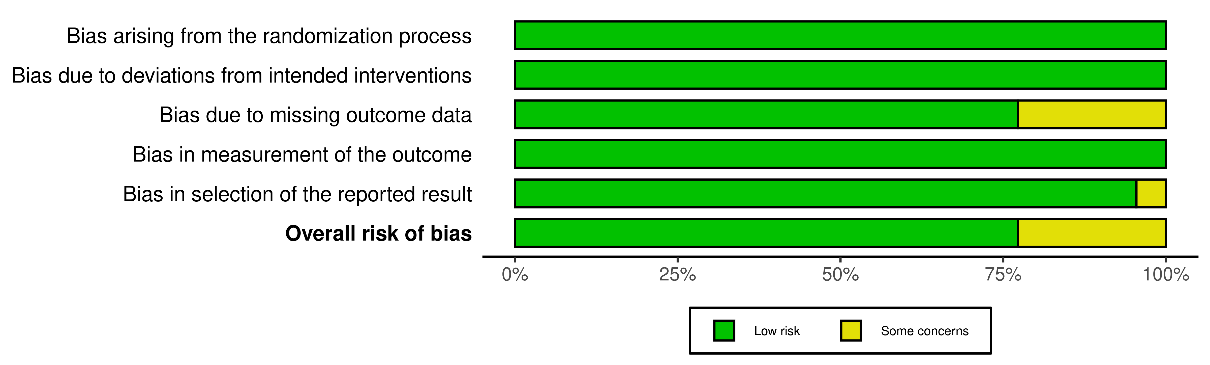


**Induction Therapy**

*Clinical remission*

Supplementary Table 3. league table showing comparative efficacy of biologics and small molecules for *induction of clinical remission – induction therapy.*

| Upadacitinib | . | . | . | . | . | . | . | . | . | . | . | . | **9.43 (5.36 -16.61)** |
| --- | --- | --- | --- | --- | --- | --- | --- | --- | --- | --- | --- | --- | --- |
| 1.90 (0.95 - 3.82) | Infliximab 5 mg | 1.10 (0.46 - 2.64) | . | . | . | . | . | **1.88 (1.18 - 3.00)** | . | . | . | . | **4.71 (3.01 - 7.38)** |
| 2.26 (0.84 - 6.11) | 1.19 (0.54 - 2.64) | Infliximab 3.5 mg | . | . | . | . | . | . | . | . | . | . | **3.73 (1.42 - 9.81)** |
| **2.68 (1.16 - 6.20)** | 1.41 (0.67 - 2.96) | 1.18 (0.42 - 3.29) | Ozanimod | . | . | . | . | . | . | . | . | . | **3.52 (1.90 - 6.55)** |
| **2.76 (1.30 - 5.88)** | 1.45 (0.76 - 2.77) | 1.22 (0.47 - 3.18) | 1.03 (0.46 - 2.29) | Guselkumab | . | . | . | . | . | . | . | . | **3.42 (2.07 - 5.65)** |
| **2.89 (1.28 - 6.51)** | 1.52 (0.74 - 3.09) | 1.28 (0.47 - 3.48) | 1.08 (0.46 - 2.53) | 1.05 (0.48 - 2.26) | Ustekinumab | . | . | . | . | . | . | . | **3.27 (1.82 - 5.85)** |
| **2.95 (1.31 - 6.61)** | 1.55 (0.76 - 3.14) | 1.30 (0.48 - 3.53) | 1.10 (0.47 - 2.57) | 1.07 (0.50 - 2.29) | 1.02 (0.45 - 2.32) | tofacitinib 10 mg | . | . | . | . | . | . | **3.20 (1.80 - 5.70)** |
| **2.97 (1.29 - 6.82)** | 1.56 (0.75 - 3.25) | 1.31 (0.47 - 3.63) | 1.11 (0.47 - 2.65) | 1.08 (0.49 - 2.37) | 1.03 (0.44 - 2.39) | 1.01 (0.44 - 2.33) | Golimumab | . | . | . | . | . | **3.18 (1.73 - 5.84)** |
| **3.39 (1.57 - 7.32)** | **1.78 (1.17 - 2.72)** | 1.50 (0.62 - 3.63) | 1.27 (0.56 - 2.85) | 1.23 (0.60 - 2.53) | 1.17 (0.54 - 2.57) | 1.15 (0.53 - 2.50) | 1.14 (0.51 - 2.55) | Etrolizumab | . | . | . | . | **3.36 (1.41 - 8.05)** |
| **3.97 (1.79 - 8.79)** | **2.09 (1.05 - 4.16)** | 1.75 (0.65 - 4.71) | 1.48 (0.64 - 3.42) | 1.44 (0.68 - 3.05) | 1.37 (0.61 - 3.08) | 1.35 (0.60 - 3.01) | 1.34 (0.59 - 3.05) | 1.17 (0.55 - 2.51) | Vedolizumab | . | . | . | **2.38 (1.36 - 4.15)** |
| **4.07 (1.91 - 8.65)** | **2.14 (1.12 - 4.08)** | 1.80 (0.69 - 4.67) | 1.52 (0.69 - 3.37) | 1.47 (0.73 - 2.99) | 1.41 (0.65 - 3.04) | 1.38 (0.64 - 2.96) | 1.37 (0.62 - 3.01) | 1.20 (0.58 - 2.46) | 1.02 (0.48 - 2.17) | Filgotinib | . | . | **2.32 (1.41 - 3.82)** |
| **4.52 (2.28 - 8.96)** | **2.38 (1.35 - 4.16)** | 2.00 (0.81 - 4.92) | 1.69 (0.81 - 3.50) | 1.64 (0.87 - 3.08) | 1.56 (0.78 - 3.15) | 1.53 (0.77 - 3.07) | 1.52 (0.74 - 3.13) | 1.33 (0.70 - 2.54) | 1.14 (0.58 - 2.24) | 1.11 (0.59 - 2.09) | Mirikizumab | . | **2.09 (1.42 - 3.07)** |
| **5.37 (2.69 -10.74)** | **2.82 (1.59 - 5.00)** | 2.37 (0.96 - 5.88) | 2.01 (0.96 - 4.20) | **1.95 (1.02 - 3.70)** | 1.86 (0.92 - 3.77) | 1.82 (0.90 - 3.68) | 1.81 (0.87 - 3.75) | 1.58 (0.82 - 3.05) | 1.35 (0.68 - 2.69) | 1.32 (0.70 - 2.50) | 1.19 (0.68 - 2.07) | Adalimumab | **1.76 (1.18 - 2.62)** |
| **9.43 (5.36 -16.61)** | **4.96 (3.29 - 7.46)** | **4.17 (1.84 - 9.41)** | **3.52 (1.90 - 6.55)** | **3.42 (2.07 - 5.65)** | **3.27 (1.82 - 5.85)** | **3.20 (1.80 - 5.70)** | **3.18 (1.73 - 5.84)** | **2.78 (1.65 - 4.67)** | **2.38 (1.36 - 4.15)** | **2.32 (1.41 - 3.82)** | **2.09 (1.42 - 3.07)** | **1.76 (1.18 - 2.62)** | Placebo |

The statistically significant results (p<0.05) are marked in red and bolded. The results of direct comparisons are presented above the labels; NMA results are shown below the labels

ADA – adalimumab; GLM – golimumab; INF – infliximab; MIC – mirikizumab; UST – ustekinumab; VDZ – vedolizumab; GUS – guselkumab; FIL – filgotinib; TOF – tofacitinib; ETR – etrolizumab; AZA – azathioprine; UPA – upadacitinib; OZA - ozanimod

Supplementary Figure S2. P-scores table (A) indicating relative ranking of biologics and small molecules as part of clinical remission - induction therapy with forest plot (B)

A B


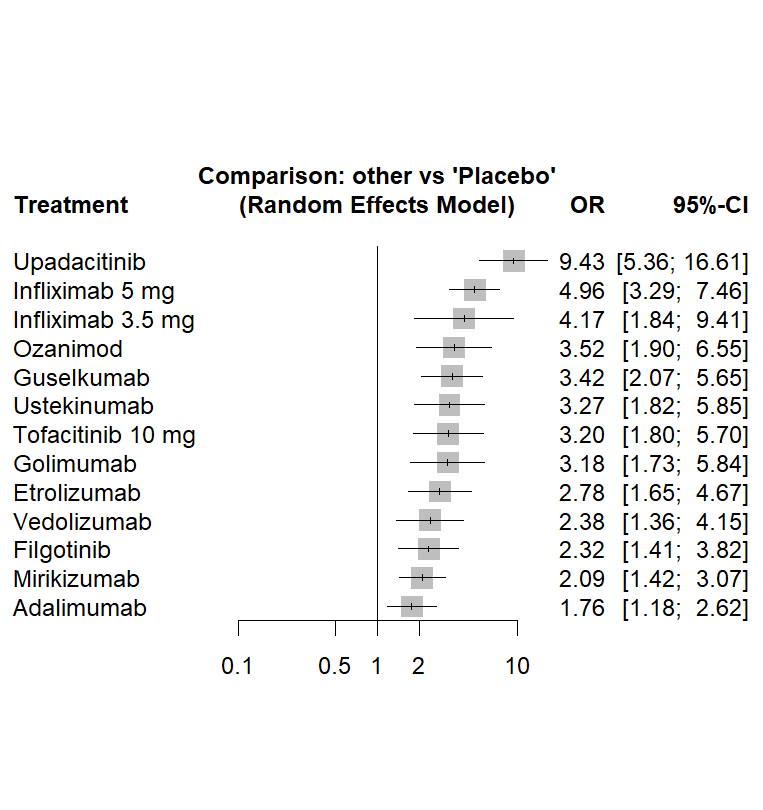


| **Clinical remission** | **P-Score** | **Rank** |
| --- | --- | --- |
| Upadacitinib | 0.9894 | 1 |
| Infliximab | 0.769 | 2 |
| Infliximab 3.5 mg | 0.6612 | 3 |
| Ozanimod | 0.633 | 4 |
| Guselkumab | 0.6193 | 5 |
| Tofacitinib 10 mg | 0.59 | 6 |
| Ustekinumab | 0.5861 | 7 |
| Golimumab | 0.5675 | 8 |
| Etrolizumab | 0.3985 | 9 |
| Vedolizumab | 0.3734 | 10 |
| Filgotinib | 0.3543 | 11 |
| Mirikizumab | 0.2833 | 12 |
| Adalimumab | 0.1731 | 13 |
| Placebo | 0.0018 | 14 |

*Clinical Response*

Supplementary Table 4. league table showing comparative efficacy of biologics and small molecules for induction of clinical response – induction therapy.

| Upadacitinib | . | . | . | . | . | . | . | . | . | . | . | . | . | . | **7.85 (5.41 -11.38)** |
| --- | --- | --- | --- | --- | --- | --- | --- | --- | --- | --- | --- | --- | --- | --- | --- |
| 1.32 (0.52 - 3.35) | IFX/AZA | . | 1.51 (0.69 - 3.29) | . | . | . | . | . | . | . | . | . | **3.33 (1.56 - 7.12)** | . | . |
| **1.90 (1.06 - 3.40)** | 1.44 (0.55 - 3.77) | Guselkumab | . | . | . | . | . | . | . | . | . | . | . | . | **4.14 (2.64 - 6.49)** |
| **1.99 (1.19 - 3.33)** | 1.51 (0.69 - 3.29) | 1.05 (0.59 - 1.86) | Infliximab 5 mg | 1.30 (0.45 - 3.76) | . | . | . | . | . | . | 1.49 (0.90 - 2.46) | . | **2.21 (1.07 - 4.57)** | . | **4.37 (2.92 - 6.53)** |
| 2.01 (0.76 - 5.32) | 1.53 (0.46 - 5.04) | 1.06 (0.39 - 2.90) | 1.01 (0.41 - 2.50) | Infliximab 3.5 mg | . | . | . | . | . | . | . | . | . | . | **4.73 (1.76 -12.70)** |
| **2.21 (1.24 - 3.97)** | 1.68 (0.64 - 4.41) | 1.17 (0.62 - 2.21) | 1.11 (0.63 - 1.97) | 1.10 (0.40 - 3.00) | Ustekinumab | . | . | . | . | . | . | . | . | . | **3.54 (2.26 - 5.55)** |
| **2.36 (1.38 - 4.06)** | 1.79 (0.70 - 4.59) | 1.25 (0.69 - 2.26) | 1.19 (0.70 - 2.01) | 1.17 (0.44 - 3.13) | 1.07 (0.59 - 1.94) | Filgotinib | . | . | . | . | . | . | . | . | **3.32 (2.24 - 4.91)** |
| **2.57 (1.51 - 4.36)** | 1.94 (0.76 - 4.95) | 1.35 (0.75 - 2.44) | 1.29 (0.77 - 2.16) | 1.27 (0.48 - 3.37) | 1.16 (0.64 - 2.08) | 1.09 (0.63 - 1.87) | tofacitinib 10 mg | . | . | . | . | . | . | . | **3.06 (2.10 - 4.46)** |
| **3.00 (1.64 - 5.48)** | 2.27 (0.86 - 6.04) | 1.58 (0.82 - 3.04) | 1.51 (0.83 - 2.72) | 1.49 (0.54 - 4.11) | 1.36 (0.71 - 2.60) | 1.27 (0.69 - 2.34) | 1.17 (0.64 - 2.14) | Ozanimod | . | . | . | . | . | . | **2.61 (1.63 - 4.20)** |
| **3.28 (1.79 - 6.00)** | 2.48 (0.93 - 6.61) | 1.73 (0.90 - 3.33) | 1.64 (0.91 - 2.98) | 1.63 (0.59 - 4.50) | 1.48 (0.77 - 2.85) | 1.39 (0.75 - 2.57) | 1.28 (0.69 - 2.35) | 1.09 (0.56 - 2.14) | Golimumab | . | . | . | . | . | **2.40 (1.49 - 3.86)** |
| **3.29 (1.89 - 5.73)** | 2.49 (0.97 - 6.44) | 1.74 (0.95 - 3.19) | 1.65 (0.96 - 2.84) | 1.64 (0.61 - 4.38) | 1.49 (0.81 - 2.73) | 1.39 (0.79 - 2.45) | 1.28 (0.74 - 2.24) | 1.10 (0.59 - 2.05) | 1.01 (0.54 - 1.89) | Mirikizumab | . | . | . | . | **2.38 (1.58 - 3.59)** |
| **3.48 (1.98 - 6.13)** | **2.64 (1.10 - 6.35)** | 1.84 (0.99 - 3.42) | **1.75 (1.16 - 2.63)** | 1.73 (0.66 - 4.53) | 1.57 (0.85 - 2.92) | 1.47 (0.83 - 2.63) | 1.36 (0.77 - 2.40) | 1.16 (0.61 - 2.19) | 1.06 (0.56 - 2.02) | 1.06 (0.59 - 1.91) | Etrolizumab | . | . | . | **1.83 (1.04 - 3.24)** |
| **4.00 (2.29 - 6.99)** | **3.03 (1.17 - 7.84)** | **2.11 (1.15 - 3.89)** | **2.01 (1.16 - 3.47)** | 1.99 (0.74 - 5.34) | 1.81 (0.98 - 3.33) | 1.69 (0.96 - 2.99) | 1.56 (0.89 - 2.73) | 1.33 (0.71 - 2.50) | 1.22 (0.65 - 2.30) | 1.22 (0.68 - 2.18) | 1.15 (0.63 - 2.08) | Vedolizumab | . | . | **1.96 (1.29 - 2.97)** |
| **4.40 (1.80 -10.73)** | **3.33 (1.56 - 7.12)** | 2.32 (0.92 - 5.86) | **2.21 (1.07 - 4.57)** | 2.19 (0.68 - 6.99) | 1.99 (0.79 - 5.02) | 1.86 (0.76 - 4.58) | 1.71 (0.70 - 4.19) | 1.47 (0.57 - 3.75) | 1.34 (0.52 - 3.44) | 1.34 (0.54 - 3.31) | 1.26 (0.55 - 2.91) | 1.10 (0.44 - 2.73) | Azathioprine | . | . |
| **4.49 (2.82 - 7.16)** | **3.40 (1.38 - 8.38)** | **2.37 (1.40 - 4.03)** | **2.26 (1.43 - 3.55)** | 2.23 (0.87 - 5.71) | **2.03 (1.19 - 3.45)** | **1.90 (1.17 - 3.08)** | **1.75 (1.09 - 2.80)** | 1.50 (0.86 - 2.60) | 1.37 (0.79 - 2.39) | 1.36 (0.83 - 2.24) | 1.29 (0.77 - 2.15) | 1.12 (0.68 - 1.85) | 1.02 (0.43 - 2.41) | Adalimumab | **1.75 (1.32 - 2.31)** |
| **7.85 (5.41 -11.38)** | **5.94 (2.53 -13.98)** | **4.14 (2.64 - 6.49)** | **3.94 (2.76 - 5.62)** | **3.90 (1.59 - 9.56)** | **3.54 (2.26 - 5.55)** | **3.32 (2.24 - 4.91)** | **3.06 (2.10 - 4.46)** | **2.61 (1.63 - 4.20)** | **2.40 (1.49 - 3.86)** | **2.38 (1.58 - 3.59)** | **2.25 (1.47 - 3.45)** | **1.96 (1.29 - 2.97)** | 1.78 (0.79 - 4.01) | **1.75 (1.32 - 2.31)** | Placebo |

The statistically significant results (p<0.05) are marked in red and bolded. The results of direct comparisons are presented above the labels; NMA results are shown below the labels

ADA – adalimumab; GLM – golimumab; INF – infliximab; MIC – mirikizumab; UST – ustekinumab; VDZ – vedolizumab; GUS – guselkumab; FIL – filgotinib; TOF – tofacitinib; ETR – etrolizumab; AZA – azathioprine; UPA – upadacitinib; OZA - ozanimod

Supplementary Figure S3. P-scores table (A) indicating relative ranking of biologics and small molecules as part of clinical response - induction therapy with forest plot (B) and network plot (C)


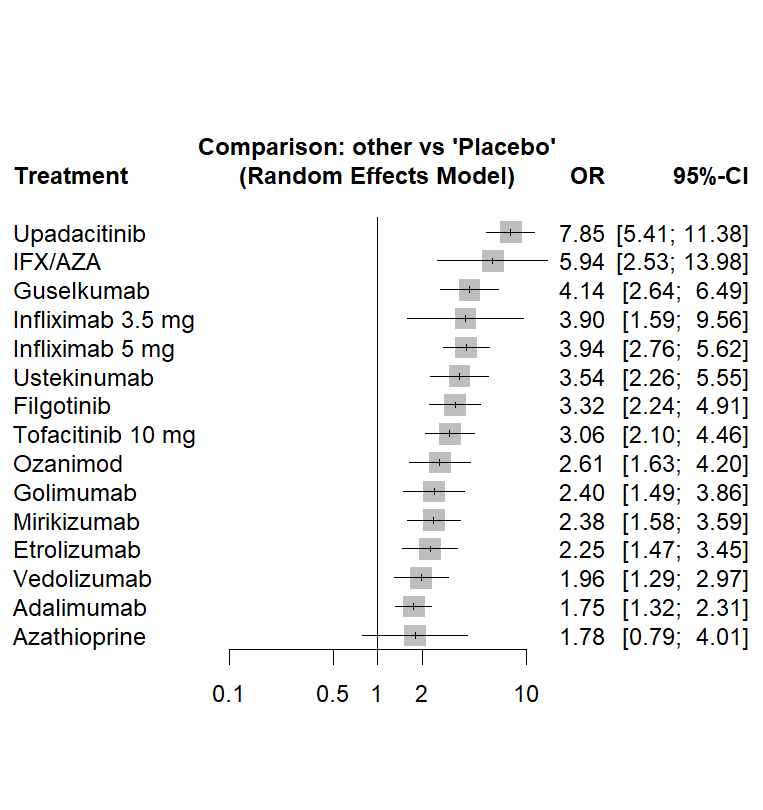
 A B

| **Clinical remission** | **P-Score** | **Rank** |
| --- | --- | --- |
| Upadacitinib | 0.9894 | 1 |
| Infliximab | 0.769 | 2 |
| Infliximab 3.5 mg | 0.6612 | 3 |
| Ozanimod | 0.633 | 4 |
| Guselkumab | 0.6193 | 5 |
| Tofacitinib 10 mg | 0.59 | 6 |
| Ustekinumab | 0.5861 | 7 |
| Golimumab | 0.5675 | 8 |
| Etrolizumab | 0.3985 | 9 |
| Vedolizumab | 0.3734 | 10 |
| Filgotinib | 0.3543 | 11 |
| Mirikizumab | 0.2833 | 12 |
| Adalimumab | 0.1731 | 13 |
| Placebo | 0.0018 | 14 |


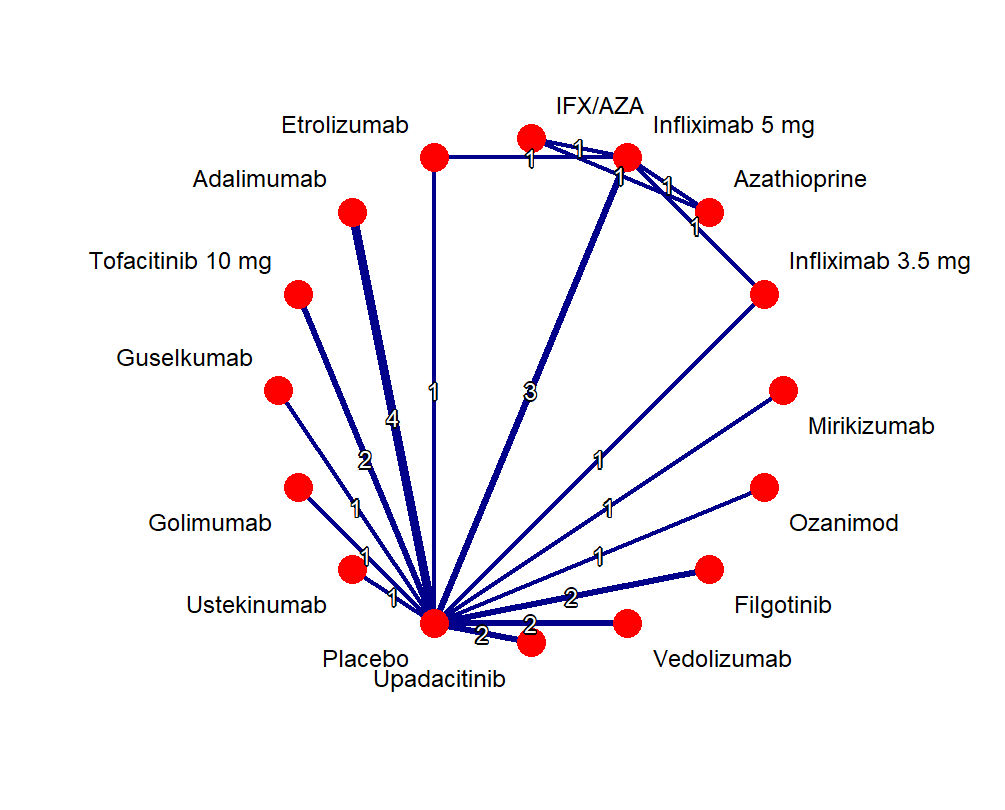


C

*Endoscopic Improvement*

Supplementary Table 5. league table showing comparative efficacy of biologics and small molecules for induction of endoscopic improvement – induction therapy.

| Upadacitinib | . | . | . | . | . | . | . | . | . | . | . | . | . | . | **8.25 (5.33 -12.77)** |
| --- | --- | --- | --- | --- | --- | --- | --- | --- | --- | --- | --- | --- | --- | --- | --- |
| 1.84 (0.80 - 4.23) | IFX/AZA | 1.41 (0.74 - 2.68) | . | . | . | . | . | . | . | . | . | . | **2.90 (1.51 - 5.57)** | . | **.** |
| **2.59 (1.52 - 4.41)** | 1.41 (0.74 - 2.68) | Infliximab 5 mg | 1.10 (0.46 - 2.65) | . | . | . | . | . | . | . | . | **1.69 (1.13 - 2.53)** | **2.06 (1.08 - 3.93)** | . | **3.42 (2.42 - 4.83)** |
| **2.49 (1.01 - 6.17)** | 1.36 (0.49 - 3.74) | 0.96 (0.44 - 2.11) | Infliximab 3.5 mg | . | . | . | . | . | . | . | . | . | . | . | **3.96 (1.54 -10.17)** |
| **2.80 (1.52 - 5.17)** | 1.52 (0.66 - 3.50) | 1.08 (0.64 - 1.84) | 1.12 (0.46 - 2.77) | Guselkumab | . | . | . | . | . | . | . | . | . | . | **2.95 (1.91 - 4.53)** |
| **2.88 (1.52 - 5.46)** | 1.57 (0.67 - 3.67) | 1.11 (0.64 - 1.95) | 1.15 (0.46 - 2.90) | 1.03 (0.54 - 1.94) | Ozanimod | . | . | . | . | . | . | . | . | . | **2.87 (1.79 - 4.57)** |
| **3.07 (1.70 - 5.54)** | 1.67 (0.74 - 3.77) | 1.18 (0.72 - 1.96) | 1.23 (0.51 - 2.99) | 1.09 (0.61 - 1.97) | 1.06 (0.58 - 1.97) | tofacitinib 10 mg | . | . | . | . | . | . | . | . | **2.69 (1.80 - 4.01)** |
| **3.57 (1.97 - 6.46)** | 1.94 (0.86 - 4.39) | 1.38 (0.83 - 2.28) | 1.43 (0.59 - 3.48) | 1.27 (0.71 - 2.30) | 1.24 (0.67 - 2.29) | 1.16 (0.66 - 2.05) | Ustekinumab | . | . | . | . | . | . | . | **2.31 (1.55 - 3.46)** |
| **3.85 (2.13 - 6.97)** | 2.10 (0.93 - 4.74) | 1.49 (0.90 - 2.47) | 1.54 (0.63 - 3.76) | 1.38 (0.76 - 2.48) | 1.34 (0.72 - 2.48) | 1.26 (0.71 - 2.21) | 1.08 (0.61 - 1.91) | Filgotinib | . | . | . | . | . | . | **2.14 (1.43 - 3.20)** |
| **3.87 (2.26 - 6.62)** | 2.11 (0.97 - 4.58) | 1.50 (0.97 - 2.32) | 1.55 (0.66 - 3.64) | 1.38 (0.81 - 2.35) | 1.34 (0.77 - 2.36) | 1.26 (0.76 - 2.10) | 1.09 (0.65 - 1.81) | 1.01 (0.60 - 1.67) | Mirikizumab | . | . | . | . | . | **2.13 (1.56 - 2.91)** |
| **4.53 (2.55 - 8.03)** | **2.46 (1.11 - 5.49)** | **1.75 (1.08 - 2.83)** | 1.82 (0.76 - 4.36) | 1.62 (0.92 - 2.85) | 1.57 (0.87 - 2.86) | 1.48 (0.86 - 2.55) | 1.27 (0.74 - 2.19) | 1.18 (0.68 - 2.03) | 1.17 (0.72 - 1.90) | Golimumab | . | . | . | . | **1.82 (1.26 - 2.64)** |
| **4.73 (2.69 - 8.31)** | **2.57 (1.16 - 5.70)** | **1.83 (1.14 - 2.92)** | 1.90 (0.80 - 4.53) | 1.69 (0.97 - 2.96) | 1.64 (0.91 - 2.96) | 1.54 (0.90 - 2.64) | 1.33 (0.78 - 2.27) | 1.23 (0.72 - 2.10) | 1.22 (0.76 - 1.96) | 1.04 (0.63 - 1.75) | Vedolizumab | . | . | . | **1.74 (1.22 - 2.49)** |
| **4.80 (2.71 - 8.50)** | **2.61 (1.27 - 5.39)** | **1.86 (1.33 - 2.60)** | 1.93 (0.83 - 4.44) | 1.72 (0.97 - 3.02) | 1.67 (0.92 - 3.02) | 1.57 (0.91 - 2.70) | 1.35 (0.78 - 2.32) | 1.25 (0.72 - 2.15) | 1.24 (0.77 - 2.01) | 1.06 (0.63 - 1.79) | 1.02 (0.61 - 1.69) | Etrolizumab | . | . | 1.48 (0.89 - 2.46) |
| **5.32 (2.30 -12.31)** | **2.90 (1.51 - 5.57)** | **2.06 (1.08 - 3.93)** | 2.13 (0.77 - 5.91) | 1.90 (0.82 - 4.38) | 1.85 (0.79 - 4.35) | 1.74 (0.77 - 3.94) | 1.49 (0.66 - 3.39) | 1.38 (0.61 - 3.14) | 1.38 (0.63 - 3.00) | 1.18 (0.53 - 2.63) | 1.13 (0.51 - 2.50) | 1.11 (0.53 - 2.30) | Azathioprine | . | . |
| **5.43 (3.26 - 9.04)** | **2.95 (1.38 - 6.31)** | **2.10 (1.40 - 3.14)** | 2.18 (0.94 - 5.02) | 1.94 (1.17 - 3.21) | **1.88 (1.10 - 3.23)** | **1.77 (1.10 - 2.86)** | 1.52 (0.94 - 2.46) | 1.41 (0.87 - 2.28) | 1.40 (0.93 - 2.11) | 1.20 (0.76 - 1.89) | 1.15 (0.74 - 1.79) | 1.13 (0.72 - 1.78) | 1.02 (0.48 - 2.19) | Adalimumab | **1.52 (1.17 - 1.98)** |
| **8.25 (5.33 -12.77)** | **4.49 (2.21 - 9.14)** | **3.19 (2.35 - 4.33)** | **3.31 (1.50 - 7.32)** | **2.95 (1.91 - 4.53)** | **2.87 (1.79 - 4.57)** | **2.69 (1.80 - 4.01)** | **2.31 (1.55 - 3.46)** | **2.14 (1.43 - 3.20)** | **2.13 (1.56 - 2.91)** | **1.82 (1.26 - 2.64)** | **1.74 (1.22 - 2.49)** | **1.72 (1.19 - 2.48)** | 1.55 (0.76 - 3.17) | **1.52 (1.17 - 1.98)** | Placebo |

The statistically significant results (p<0.05) are marked in red and bolded. The results of direct comparisons are presented above the labels; NMA results are shown below the labels

ADA – adalimumab; GLM – golimumab; INF – infliximab; MIC – mirikizumab; UST – ustekinumab; VDZ – vedolizumab; GUS – guselkumab; FIL – filgotinib; TOF – tofacitinib; ETR – etrolizumab; AZA – azathioprine; UPA – upadacitinib; OZA - ozanimod

Supplementary Figure S4. P-scores table (A) indicating relative ranking of biologics and small molecules as part of endoscopic improvement - induction therapy with forest plot (B) and network plot (C)


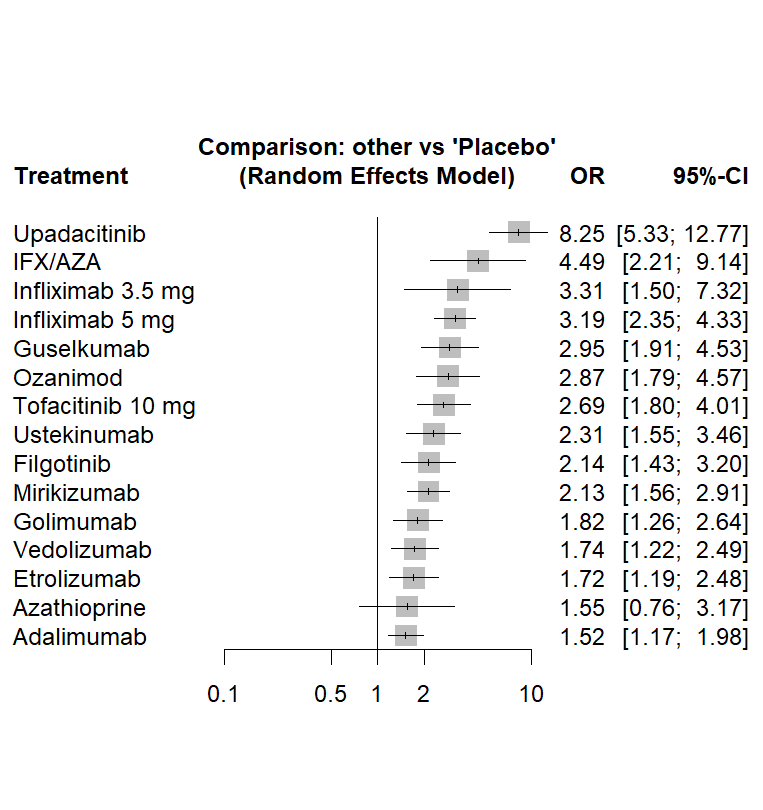


A B

| **Clinical remission** | **P-Score** | **Rank** |
| --- | --- | --- |
| Upadacitinib | 0.9894 | 1 |
| Infliximab | 0.769 | 2 |
| Infliximab 3.5 mg | 0.6612 | 3 |
| Ozanimod | 0.633 | 4 |
| Guselkumab | 0.6193 | 5 |
| Tofacitinib 10 mg | 0.59 | 6 |
| Ustekinumab | 0.5861 | 7 |
| Golimumab | 0.5675 | 8 |
| Etrolizumab | 0.3985 | 9 |
| Vedolizumab | 0.3734 | 10 |
| Filgotinib | 0.3543 | 11 |
| Mirikizumab | 0.2833 | 12 |
| Adalimumab | 0.1731 | 13 |
| Placebo | 0.0018 | 14 |

C


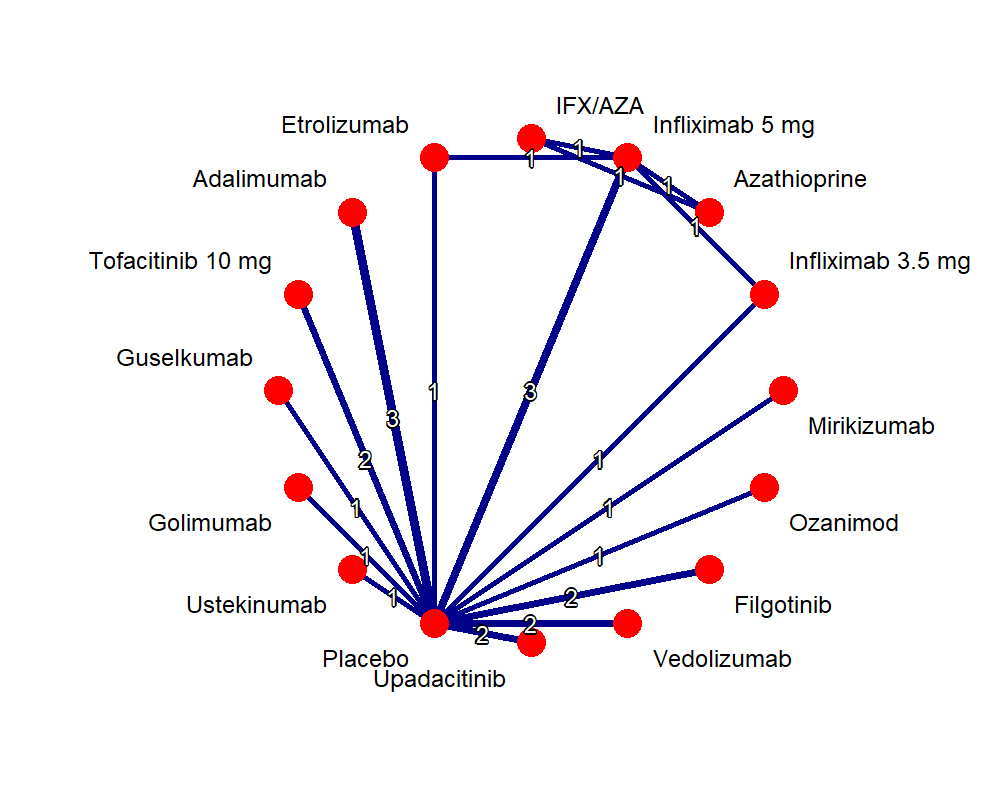


**Maintenance Therapy**

*Clinical remission*

Supplementary Table 6. league table showing comparative efficacy of biologics and small molecules for maintenance of clinical remission – Maintenance therapy

| Upadacitinib | . | . | . | . | . | . | . | . | . | . | . | **7.87 (3.90 -15.88)** |
| --- | --- | --- | --- | --- | --- | --- | --- | --- | --- | --- | --- | --- |
| 1.50 (0.50 - 4.48) | Filgotinib | . | . | . | . | . | . | . | . | . | . | **5.25 (2.27 -12.16)** |
| 1.84 (0.73 - 4.64) | 1.23 (0.44 - 3.45) | Guselkumab | . | . | . | . | . | . | . | . | . | **4.28 (2.34 - 7.81)** |
| 1.88 (0.72 - 4.92) | 1.25 (0.43 - 3.65) | 1.02 (0.42 - 2.49) | tofacitinib | . | . | . | . | . | . | . | . | **4.18 (2.17 - 8.08)** |
| 2.05 (0.91 - 4.63) | 1.37 (0.54 - 3.49) | 1.11 (0.54 - 2.32) | 1.09 (0.50 - 2.37) | Vedolizumab | . | . | . | 1.57 (0.95 - 2.60) | . | . | . | **3.67 (2.23 - 6.05)** |
| **2.66 (1.09 - 6.50)** | 1.77 (0.65 - 4.85) | 1.44 (0.64 - 3.27) | 1.41 (0.60 - 3.34) | 1.30 (0.65 - 2.59) | Mirikizumab | . | . | . | . | . | . | **2.96 (1.70 - 5.15)** |
| **2.83 (1.10 - 7.27)** | 1.89 (0.66 - 5.39) | 1.54 (0.64 - 3.68) | 1.50 (0.60 - 3.74) | 1.38 (0.65 - 2.94) | 1.06 (0.46 - 2.46) | Golimumab | . | . | . | . | . | **2.78 (1.48 - 5.23)** |
| **3.05 (1.23 - 7.57)** | 2.03 (0.73 - 5.64) | 1.66 (0.72 - 3.82) | 1.62 (0.67 - 3.89) | 1.49 (0.73 - 3.03) | 1.15 (0.52 - 2.55) | 1.08 (0.46 - 2.53) | Ozanimod | . | . | . | . | **2.58 (1.45 - 4.60)** |
| **3.07 (1.35 - 7.01)** | 2.05 (0.80 - 5.27) | 1.67 (0.79 - 3.51) | 1.63 (0.74 - 3.59) | 1.50 (0.99 - 2.27) | 1.16 (0.57 - 2.33) | 1.09 (0.50 - 2.33) | 1.01 (0.49 - 2.08) | Adalimumab | . | . | . | **2.70 (1.56 - 4.67)** |
| **3.19 (1.27 - 8.04)** | 2.13 (0.76 - 5.98) | 1.74 (0.74 - 4.06) | 1.70 (0.70 - 4.14) | 1.56 (0.75 - 3.23) | 1.20 (0.53 - 2.72) | 1.13 (0.47 - 2.70) | 1.05 (0.46 - 2.41) | 1.04 (0.50 - 2.18) | Ustekinumab | . | . | **2.46 (1.35 - 4.49)** |
| **3.58 (1.44 - 8.88)** | 2.39 (0.86 - 6.61) | 1.95 (0.85 - 4.48) | 1.90 (0.79 - 4.56) | 1.75 (0.86 - 3.55) | 1.35 (0.61 - 3.00) | 1.27 (0.54 - 2.97) | 1.17 (0.52 - 2.66) | 1.17 (0.57 - 2.40) | 1.12 (0.49 - 2.57) | Infliximab | 1.24 (0.67 - 2.29) | **2.68 (1.30 - 5.52)** |
| **5.12 (2.06 -12.73)** | **3.42 (1.23 - 9.48)** | **2.78 (1.21 - 6.42)** | **2.72 (1.13 - 6.55)** | **2.50 (1.23 - 5.10)** | 1.93 (0.86 - 4.30) | 1.81 (0.77 - 4.26) | 1.68 (0.74 - 3.81) | 1.67 (0.81 - 3.44) | 1.60 (0.70 - 3.69) | 1.43 (0.84 - 2.42) | Etrolizumab | 1.25 (0.60 - 2.60) |
| **7.87 (3.90 -15.88)** | **5.25 (2.27 -12.16)** | **4.28 (2.34 - 7.81)** | **4.18 (2.17 - 8.08)** | **3.84 (2.54 - 5.81)** | **2.96 (1.70 - 5.15)** | **2.78 (1.48 - 5.23)** | **2.58 (1.45 - 4.60)** | **2.56 (1.66 - 3.95)** | **2.46 (1.35 - 4.49)** | **2.20 (1.24 - 3.91)** | 1.54 (0.86 - 2.74) | Placebo |

The statistically significant results (p<0.05) are marked in red and bolded. The results of direct comparisons are presented above the labels; NMA results are shown below the labels

ADA – adalimumab; GLM – golimumab; INF – infliximab; MIC – mirikizumab; UST – ustekinumab; VDZ – vedolizumab; GUS – guselkumab; FIL – filgotinib; TOF – tofacitinib; ETR – etrolizumab; AZA – azathioprine; UPA – upadacitinib; OZA - ozanimod

Supplementary Figure S5. P-scores table (A) indicating relative ranking of biologics and small molecules as part of clinical remission - Maintenance therapy with forest plot (B)

A B


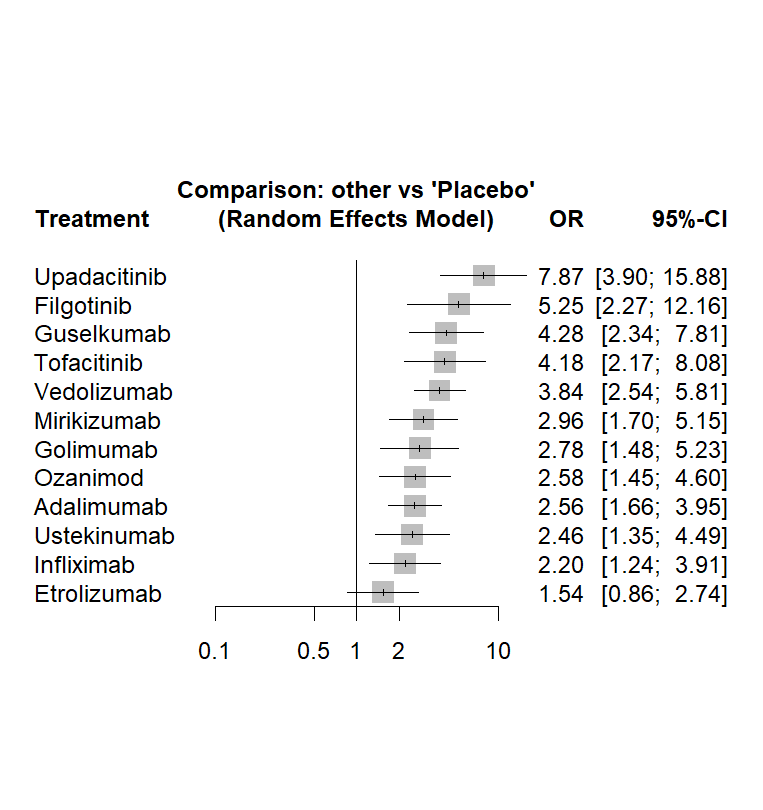


| **Clinical remission** | **P-Score** | **Rank** |
| --- | --- | --- |
| Upadacitinib | 0.9894 | 1 |
| Infliximab | 0.769 | 2 |
| Infliximab 3.5 mg | 0.6612 | 3 |
| Ozanimod | 0.633 | 4 |
| Guselkumab | 0.6193 | 5 |
| Tofacitinib 10 mg | 0.59 | 6 |
| Ustekinumab | 0.5861 | 7 |
| Golimumab | 0.5675 | 8 |
| Etrolizumab | 0.3985 | 9 |
| Vedolizumab | 0.3734 | 10 |
| Filgotinib | 0.3543 | 11 |
| Mirikizumab | 0.2833 | 12 |
| Adalimumab | 0.1731 | 13 |
| Placebo | 0.0018 | 14 |

Endoscopic Improvement

Supplementary Table 7. league table showing comparative efficacy of biologics and small molecules for maintenance of endoscopic improvement – Maintenance therapy

| Upadacitinib | . | . | . | . | . | . | . | . | . | . | . | **9.30 (5.32 -16.23)** |
| --- | --- | --- | --- | --- | --- | --- | --- | --- | --- | --- | --- | --- |
| 2.04 (0.99 - 4.20) | Guselkumab | . | . | . | . | . | . | . | . | . | . | **4.56 (2.87 - 7.23)** |
| **2.35 (1.11 - 4.99)** | 1.15 (0.58 - 2.28) | Tofacitinib | . | . | . | . | . | . | . | . | . | **3.95 (2.39 - 6.53)** |
| **2.48 (1.31 - 4.70)** | 1.22 (0.70 - 2.12) | 1.05 (0.58 - 1.90) | Vedolizumab | . | . | . | . | . | . | . | **1.72 (1.27 - 2.32)** | **4.11 (2.71 - 6.21)** |
| **2.45 (1.06 - 5.63)** | 1.20 (0.55 - 2.59) | 1.04 (0.47 - 2.31) | 0.99 (0.49 - 1.97) | Filgotinib | . | . | . | . | . | . | . | **3.80 (2.05 - 7.05)** |
| **2.69 (1.36 - 5.28)** | 1.32 (0.72 - 2.40) | 1.14 (0.61 - 2.15) | 1.08 (0.66 - 1.78) | 1.10 (0.53 - 2.27) | Mirikizumab | . | . | . | . | . | . | **3.46 (2.36 - 5.08)** |
| **2.83 (1.38 - 5.81)** | 1.39 (0.72 - 2.65) | 1.20 (0.61 - 2.37) | 1.14 (0.66 - 1.98) | 1.16 (0.54 - 2.49) | 1.05 (0.58 - 1.91) | Infliximab | . | . | 1.28 (0.83 - 1.97) | . | . | **3.75 (2.09 - 6.73)** |
| **3.55 (1.75 - 7.23)** | 1.74 (0.92 - 3.30) | 1.51 (0.77 - 2.95) | 1.43 (0.83 - 2.46) | 1.45 (0.68 - 3.10) | 1.32 (0.74 - 2.38) | 1.26 (0.67 - 2.37) | Ustekinumab | . | . | . | . | **2.62 (1.68 - 4.07)** |
| **3.81 (1.86 - 7.77)** | 1.87 (0.98 - 3.54) | 1.62 (0.83 - 3.17) | 1.53 (0.89 - 2.65) | 1.56 (0.73 - 3.33) | 1.42 (0.79 - 2.55) | 1.35 (0.71 - 2.55) | 1.07 (0.57 - 2.01) | Golimumab | . | . | . | **2.44 (1.56 - 3.82)** |
| **3.90 (1.89 - 8.02)** | **1.91 (1.00 - 3.66)** | 1.65 (0.84 - 3.27) | 1.57 (0.90 - 2.74) | 1.59 (0.74 - 3.44) | 1.45 (0.80 - 2.64) | 1.38 (0.94 - 2.02) | 1.10 (0.58 - 2.07) | 1.02 (0.54 - 1.94) | Etrolizumab | . | . | **2.08 (1.15 - 3.77)** |
| **3.98 (2.01 - 7.86)** | **1.95 (1.06 - 3.57)** | 1.69 (0.89 - 3.20) | 1.60 (0.97 - 2.65) | 1.62 (0.78 - 3.38) | 1.48 (0.85 - 2.56) | 1.41 (0.77 - 2.57) | 1.12 (0.62 - 2.02) | 1.04 (0.58 - 1.89) | 1.02 (0.56 - 1.87) | Ozanimod | . | **2.34 (1.58 - 3.46)** |
| **4.47 (2.37 - 8.41)** | **2.19 (1.26 - 3.79)** | **1.90 (1.06 - 3.41)** | **1.80 (1.38 - 2.35)** | 1.83 (0.92 - 3.63) | **1.66 (1.02 - 2.71)** | 1.58 (0.92 - 2.73) | 1.26 (0.74 - 2.14) | 1.17 (0.69 - 2.01) | 1.15 (0.66 - 1.98) | 1.12 (0.69 - 1.84) | Adalimumab | **1.94 (1.34 - 2.80)** |
| **9.30 (5.32 -16.23)** | **4.56 (2.87 - 7.23)** | **3.95 (2.39 - 6.53)** | **3.75 (2.74 - 5.12)** | **3.80 (2.05 - 7.05)** | **3.46 (2.36 - 5.08)** | **3.29 (2.08 - 5.19)** | **2.62 (1.68 - 4.07)** | **2.44 (1.56 - 3.82)** | **2.39 (1.51 - 3.78)** | **2.34 (1.58 - 3.46)** | **2.08 (1.54 - 2.81)** | Placebo |

The statistically significant results (p<0.05) are marked in red and bolded. The results of direct comparisons are presented above the labels; NMA results are shown below the labels

ADA – adalimumab; GLM – golimumab; INF – infliximab; MIC – mirikizumab; UST – ustekinumab; VDZ – vedolizumab; GUS – guselkumab; FIL – filgotinib; TOF – tofacitinib; ETR – etrolizumab; AZA – azathioprine; UPA – upadacitinib; OZA - ozanimod

Supplementary Figure S6. P-scores table (A) indicating relative ranking of biologics and small molecules as part of endoscopic improvement - Maintenance therapy with forest plot (B) and network plot (C).


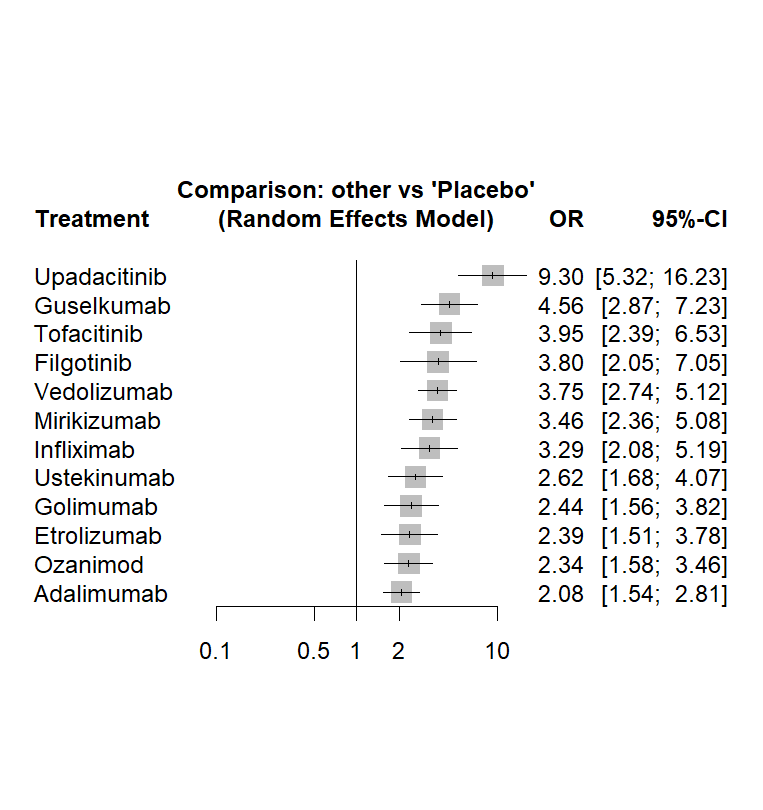


A B

| **Clinical remission** | **P-Score** | **Rank** |
| --- | --- | --- |
| Upadacitinib | 0.9894 | 1 |
| Infliximab | 0.769 | 2 |
| Infliximab 3.5 mg | 0.6612 | 3 |
| Ozanimod | 0.633 | 4 |
| Guselkumab | 0.6193 | 5 |
| Tofacitinib 10 mg | 0.59 | 6 |
| Ustekinumab | 0.5861 | 7 |
| Golimumab | 0.5675 | 8 |
| Etrolizumab | 0.3985 | 9 |
| Vedolizumab | 0.3734 | 10 |
| Filgotinib | 0.3543 | 11 |
| Mirikizumab | 0.2833 | 12 |
| Adalimumab | 0.1731 | 13 |
| Placebo | 0.0018 | 14 |


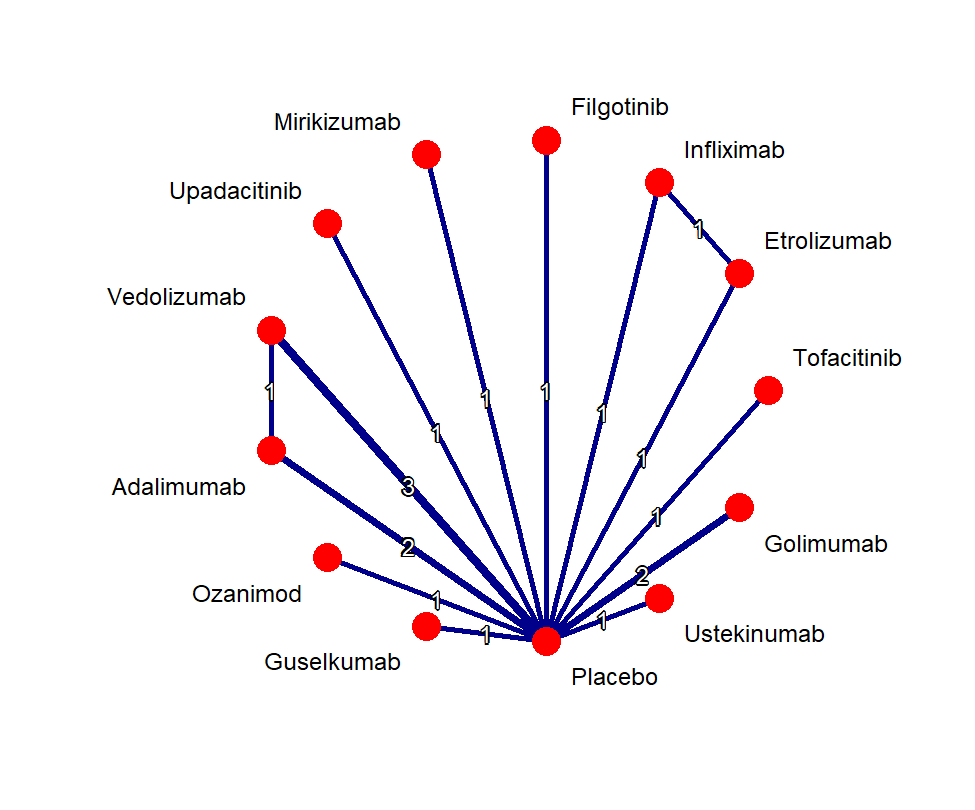


C

Steroid free remission

Supplementary Table 8. league table showing comparative efficacy of biologics and small molecules for achievement of steroid free remission – Maintenance therapy

| Tofacitinib | . | . | . | . | . | . | . | . | . | . | **10.22 (2.88 -36.32)** |
| --- | --- | --- | --- | --- | --- | --- | --- | --- | --- | --- | --- |
| 1.42 (0.31 - 6.53) | Upadacitinib | . | . | . | . | . | . | . | . | . | **7.18 (3.09 -16.71)** |
| 2.27 (0.54 - 9.49) | 1.59 (0.54 - 4.67) | Adalimumab | . | . | . | . | 1.94 (0.95 - 3.94) | . | . | . | **3.51 (1.42 - 8.67)** |
| 2.41 (0.62 - 9.29) | 1.69 (0.65 - 4.43) | 1.06 (0.47 - 2.40) | Guselkumab | . | . | . | . | . | . | . | **4.25 (2.67 - 6.75)** |
| 2.37 (0.57 - 9.81) | 1.67 (0.58 - 4.80) | 1.05 (0.42 - 2.64) | 0.98 (0.45 - 2.17) | Ustekinumab | . | . | . | . | . | . | **4.31 (2.28 - 8.17)** |
| 3.48 (0.79 -15.30) | 2.44 (0.78 - 7.64) | 1.54 (0.56 - 4.24) | 1.44 (0.59 - 3.54) | 1.47 (0.54 - 3.98) | Infliximab | . | . | . | 1.15 (0.51 - 2.60) | . | **3.56 (1.39 - 9.14)** |
| 3.49 (0.92 -13.23) | 2.45 (0.96 - 6.27) | 1.54 (0.70 - 3.37) | 1.45 (0.78 - 2.69) | 1.47 (0.69 - 3.15) | 1.00 (0.42 - 2.39) | Mirikizumab, | . | . | . | . | **2.93 (1.94 - 4.42)** |
| 3.76 (0.93 -15.22) | 2.64 (0.94 - 7.40) | 1.66 (0.91 - 3.03) | 1.56 (0.74 - 3.31) | 1.59 (0.66 - 3.78) | 1.08 (0.41 - 2.84) | 1.08 (0.53 - 2.21) | Vedolizumab | . | . | . | **3.14 (1.58 - 6.25)** |
| **4.42 (1.15 -16.95)** | **3.11 (1.20 - 8.07)** | 1.95 (0.87 - 4.35) | 1.84 (0.96 - 3.49) | 1.86 (0.86 - 4.06) | 1.27 (0.52 - 3.08) | 1.27 (0.69 - 2.32) | 1.18 (0.56 - 2.46) | Ozanimod | . | . | **2.31 (1.48 - 3.61)** |
| **4.63 (1.04 -20.67)** | **3.25 (1.02 -10.37)** | 2.04 (0.72 - 5.77) | 1.92 (0.77 - 4.83) | 1.95 (0.70 - 5.41) | 1.33 (0.66 - 2.69) | 1.33 (0.54 - 3.24) | 1.23 (0.46 - 3.31) | 1.05 (0.42 - 2.60) | Etrolizumab | . | 1.75 (0.62 - 4.93) |
| **6.45 (1.50 -27.63)** | **4.53 (1.50 -13.69)** | **2.85 (1.07 - 7.56)** | **2.68 (1.14 - 6.28)** | **2.72 (1.04 - 7.09)** | 1.85 (0.65 - 5.28) | 1.85 (0.81 - 4.21) | 1.71 (0.68 - 4.33) | 1.46 (0.63 - 3.38) | 1.39 (0.48 - 4.06) | Golimumab | 1.59 (0.78 - 3.24) |
| **10.22 (2.88 -36.32)** | **7.18 (3.09 -16.71)** | **4.51 (2.31 - 8.79)** | **4.25 (2.67 - 6.75)** | **4.31 (2.28 - 8.17)** | **2.94 (1.37 - 6.32)** | **2.93 (1.94 - 4.42)** | **2.72 (1.51 - 4.90)** | **2.31 (1.48 - 3.61)** | **2.21 (1.00 - 4.89)** | 1.59 (0.78 - 3.24) | Placebo |

Supplementary Figure S7. P-scores table (A) indicating relative ranking of biologics and small molecules as in achieving steroid free remission - Maintenance therapy with forest plot (B) and network plot (C).


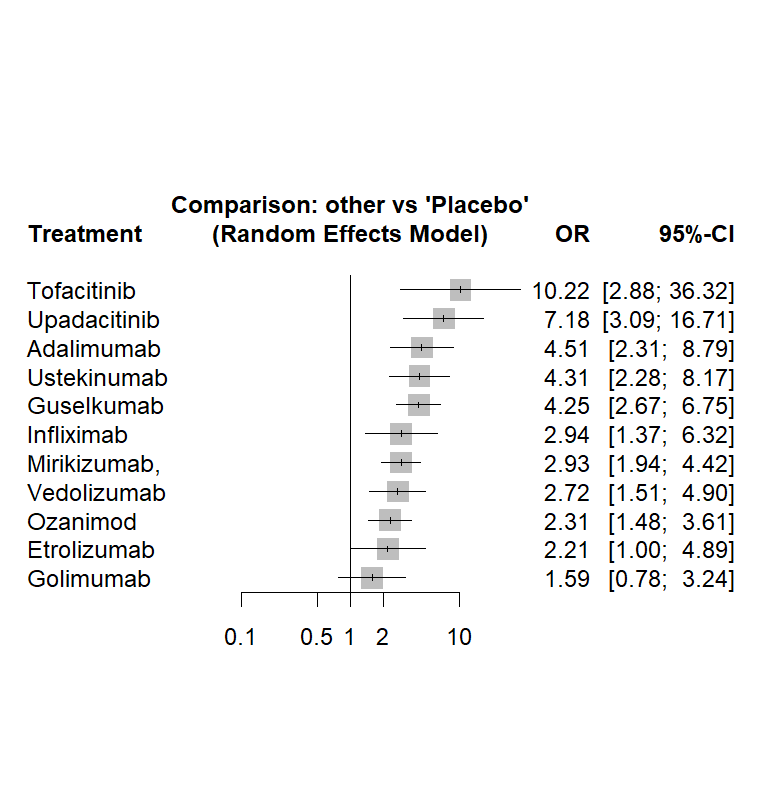
A B

| **Clinical remission** | **P-Score** | **Rank** |
| --- | --- | --- |
| Upadacitinib | 0.9894 | 1 |
| Infliximab | 0.769 | 2 |
| Infliximab 3.5 mg | 0.6612 | 3 |
| Ozanimod | 0.633 | 4 |
| Guselkumab | 0.6193 | 5 |
| Tofacitinib 10 mg | 0.59 | 6 |
| Ustekinumab | 0.5861 | 7 |
| Golimumab | 0.5675 | 8 |
| Etrolizumab | 0.3985 | 9 |
| Vedolizumab | 0.3734 | 10 |
| Filgotinib | 0.3543 | 11 |
| Mirikizumab | 0.2833 | 12 |
| Adalimumab | 0.1731 | 13 |
| Placebo | 0.0018 | 14 |

C


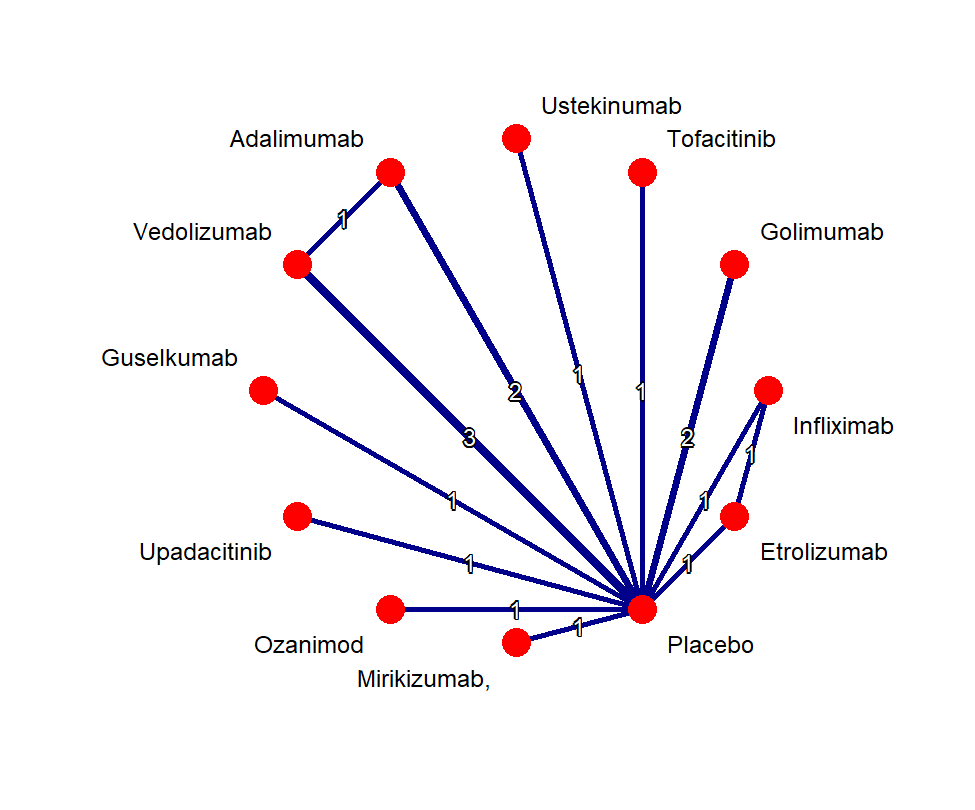


| **Endpoint** | **Tau** | **I^2^** | **Q value - total** | **P value (Q test) - total** | **Q value - within designs** | **P value**  **(Q test) – within designs** | **Q value – between designs** | **P value (Q test) - between designs** |
| --- | --- | --- | --- | --- | --- | --- | --- | --- |
| Induction phase | | | | | | | | |
| Clinical remission | 0.0524 | 1.8% | 9.16 | 0.4225 | 8.77 | 0.2695 | 0.39 | 0.8230 |
| Clinical response | 0.1575 | 28.8% | 14.04 | 0.1712 | 11.86 | 0.1575 | 2.18 | 0.3363 |
| Endoscopic improvement | 0 | 0% | 5.03 | 0.8313 | 4.04 | 0.7753 | 1 | 0.6079 |
| Maintenance phase | | | | | | | | |
| Clinical remission | 0.1979 | 24.2% | 7.91 | 0.2445 | 6.62 | 0.1573 | 1.29 | 0.5242 |
| Endoscopic improvement | 0 | 0% | 5.38 | 0.4956 | 4.45 | 0.3483 | 0.93 | 0.6273 |
| Steroid free remission | 0 | 0% | 5.35 | 0.5004 | 4.23 | 0.3763 | 1.12 | 0.5713 |
